# Topology changes of *Hydra* define actin orientation defects as organizers of morphogenesis

**DOI:** 10.1126/sciadv.adr9855

**Published:** 2025-01-17

**Authors:** Yamini Ravichandran, Matthias Vogg, Karsten Kruse, Daniel J. G. Pearce, Aurélien Roux

**Affiliations:** ^1^ Department of Biochemistry, Université de Genève, CH-1211 Genève, Switzerland.; ^2^ Department of Genetics & Evolution, Université de Genève, CH-1211 Genève, Switzerland.; ^3^ Department of Theoretical Physics, Université de Genève, CH-1211 Genève, Switzerland.

## Abstract

*Hydra* regenerates one head when cut, but how forces shaping the head are coordinated remains unclear. Soft compression of *Hydra*’s head-regenerating tissues induces the formation of viable, two-headed animals. Compression creates new topological defects in the supracellular orientational order of muscular actin fibers, associated with additional heads. Theory supports that these defects organize muscle stresses required to shape the head. By compressing head-regenerating tissues along their body axis, we formed toroidal tissues, whose unique topology allows for the absence of defects. Toroids with no actin defects did not regenerate. Toroids with actin defects regenerated into viable toroidal animals with a bifurcated body. Topological defects in the actin orientational order are thus necessary for complete regeneration of *Hydra*, defining actin topological defects as mechanical organizers of morphogenesis.

## Teaser

Defects in the alignment of muscle fibers determine the number and position of heads in regenerating freshwater *Hydra*.

## INTRODUCTION

Morphogenesis and regeneration share common principles of development that establish body axes and cell fates. However, less is known about how cells and tissues coordinate forces that shape organisms. *Hydra* is an established animal model for regeneration and developmental biology since the 18th century (*1*). The evolutionarily conserved Wnt family proteins specify the body axis in the animal by forming the head organizer complex (*2*–*5*). Up-regulation of β-catenin—a mechanosensing transducer of the Wnt pathway—leads to the formation of additional heads during regeneration, showing that head determination and tissue mechanics are coupled (*6*).

Mechanical cues such as osmotic oscillations are also essential for *Hydra* regeneration (*7*). Breaking of symmetry in these oscillations driven by muscular contractions plays a role in specifying the body axis (*8*, *9*). These contractions rely on ordered basal actin bundles that display supracellular organization, reminiscent of a liquid crystal–like nematic order: In the adult animal, they align parallel to the body axis in the ectoderm, forming aster-like topological defects at the foot and at the mouth, and in the endoderm, they form concentric circles around the body of the animal (*10*, *11*). Topological defects are singularities in the nematic order—point locations where the order is lost—surrounded by a specific pattern of order function of their topological charge. On surfaces, the distortion of the nematic field around defects is energetically coupled to curvature, an effect that can passively drive surface deformation (*12*). In living systems that behave as active materials able to generate internal forces, topological defects can generate unique patterns of stress around them, which can drive motion or deformation (*12*). Examples of defects acting as force-organizing centers include defects required for cell extrusion, cell sorting, and collective cell migration (*13*). More recently, we showed that the three-dimensional (3D) growth of cellular vortices is shaped by integer topological defects (*14*, *15*), a process similar to the germ band extension in *Drosophila* (*16*). The common mechanism behind these processes is that if considering that every muscle fiber (whether they are actin cables or cells) applies the same force per unit length, then the organization of muscle fibers around topological defects creates gradients of stresses (*14*). Because changing the shape of a material requires to apply force gradients (if the force/pressure is applied homogeneously, then the material shrinks or expands but does not change its shape), topological defects are intrinsically capable of changing the shape of tissues and even cells (*17*).

These studies highlight the possible role of defects as mechanical organizers of morphogenesis in biological systems. This concept is most supported by recent studies conducted in *Hydra* showing a strong correlation between the position of topological defects in the actin with the position of the new mouth and foot during regeneration (*10*). Although summing up the charge of topological defects across the body of the animal, they all add up to a total charge of 2, which corresponds to the preserved topological charge displayed by any closed polyhedron (*13*). Only upon changing the entire topology can one alter the total topological charge of a system, which can be performed by piercing a hole instead of simply deforming a surface (*13*).

**Fig. 1.**
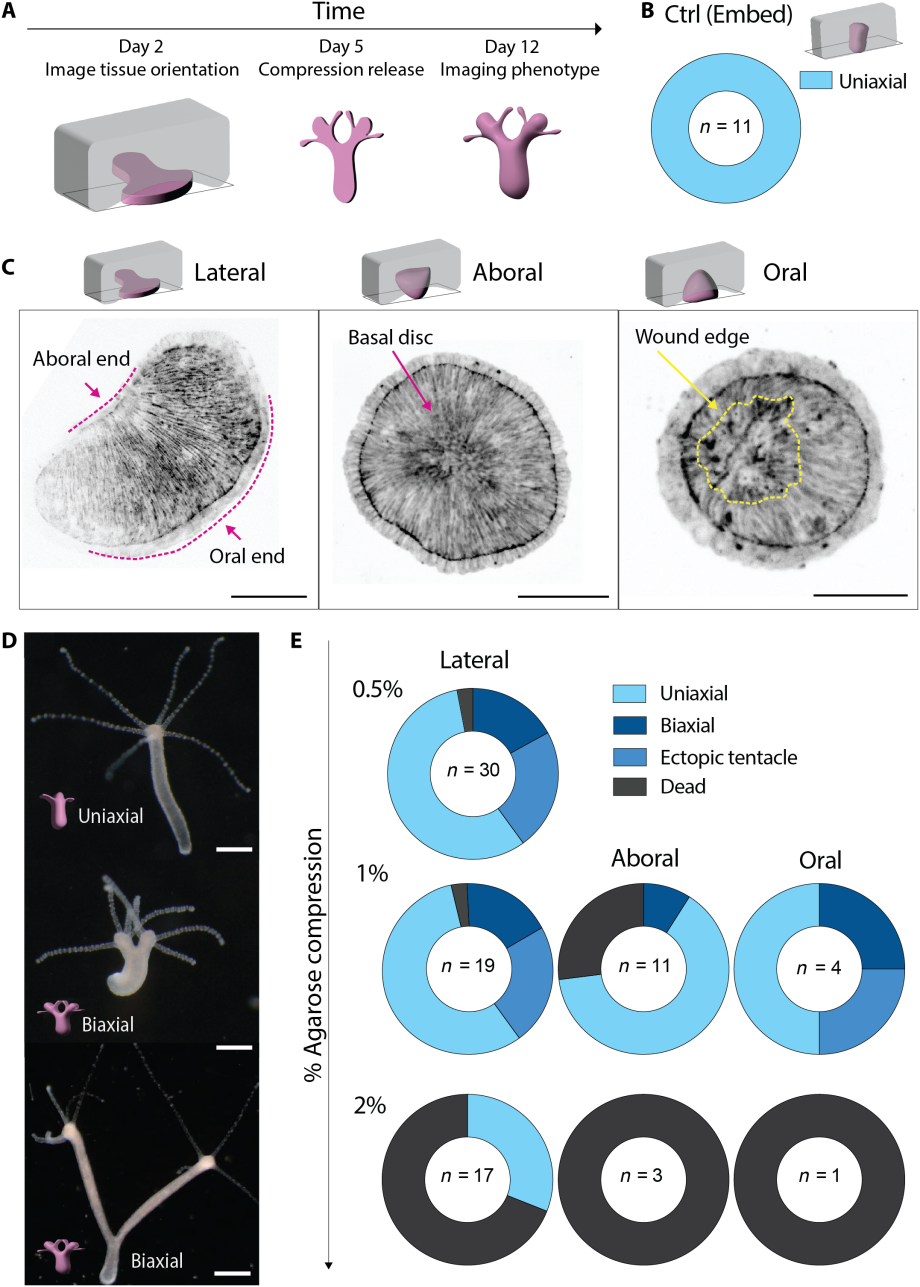
Mechanical induction of bicephalous morphogenesis in *Hydra*. (**A**) Schematics showing the experimental timeline of head-regenerating tissues under compression, release, and phenotype analysis. (**B**) Ring plot showing the phenotype distribution for control head regeneration experiments (0.5% agarose embedded) and corresponding schematic of the experimental setup. (**C**) (Top) Schematics of head-regenerating tissue orientations under compression and (Bottom) corresponding spinning disk microscopy images of GFP-Ecto-LifeAct tissues. Scale bars, 100 μm. Pink dashed lines depict the polarity of tissue contours; arrows show oral/aboral ends and basal disc; the yellow dashed line highlights the wound edge marked by a disordered actin region. (**D**) Live color images of fully regenerated *Hydra* postcompression release at 12 dpd. Scale bars, 500 μm. (**E**) Ring plots showing the distribution of phenotypes at 12 dpd postcompression release for different percentages of agarose and different orientations.

Given the nematic properties displayed by actin (*10*), we aimed at changing the nematic order in the regenerating *Hydra* to probe the role of actin defects. For this, we confined *Hydra* during head regeneration: Adult animals were sectioned in their transverse plane, and the part containing the foot, hereafter called the head-regenerating tissue, was left to recover for 6 hours. Head-regenerating tissues were then subjected to soft compression between an agarose slab of varying stiffness (0.5, 1, and 2% agarose) and a glass-bottom dish. Head-regenerating tissues were kept under compression for 4 days post-dissection (dpd) and then released on day 5 (Fig. 1A). Animals were screened at 12 dpd to allow all tissues to complete regeneration before phenotypic analysis. As a control, to confirm that agarose did not affect regeneration, head-regenerating tissues were embedded in agarose. These tissues regenerated into viable uniaxial animals within 4 dpd (Fig. 1B and movie S1).

## RESULTS

### Mechanical induction of bicephalous morphogenesis in *Hydra*

Under compression, head-regenerating tissues adopted one of the three possible orientations: lateral, oral, or aboral. In the lateral orientation, the tissue axis is perpendicular to the compression axis; oral corresponds to compression aligned with the tissue axis, the foot facing the glass; and aboral is the inverse of oral orientation (Fig. 1C). We captured the tissue orientation 12 hours after compression [24 hours post-dissection (hpd)]. We observed that, under the softest 0.5% agarose compression (0.5% AC), all head-regenerating tissues oriented laterally (fig. S1, A and E), whereas with stiffer agarose (1 and 2% AC), a significant fraction displayed oral and aboral orientations, indicating their inability to reorient under compression (fig. S1, A and E).

At 12 dpd, the most notable phenotype was a substantial proportion of bicephalous animals (30% at 0.5% AC) (Fig. 1, D and E). As the single foot is symmetrically connected to the two heads, we refer to them hereafter as biaxial animals. Uniaxial animals with ectopic tentacles were also observed (25% at 0.5% AC), similar to the ectopic tentacles observed in weak Wnt3 overexpressing mutants (fig. S1B) (*5*, *18*). However, biaxial animals are not equivalent to Wnt3 overexpressing mutants with ectopic heads as ectopic heads form after the establishment of the primary body axis marked with the primary head (*5*, *18*). The biaxial animals were viable, and the two heads functional as evidenced by an *Artemia* feeding assay (fig. S1C and movie S2). In situ hybridization of Wnt3 RNA confirmed the full differentiation of the additional heads (fig. S1D).

We observed that deformation of head-regenerating tissues increased gradually with the agarose stiffness (fig. S1E). The most notable change induced by increasing stiffness was a significant fraction of dead animals at 1 and 2% AC (Fig. 1E and movie S3). Furthermore, the initial orientation markedly changed the relative proportions of the phenotypes: Tissues oriented laterally displayed the same phenotypic distribution in regenerated animals for both 0.5 and 1% AC (Fig. 1E). However, head-regenerating tissues with oral and aboral orientations, present only in 1 and 2% AC, had a much higher proportion of death (Fig. 1E and movie S3). Thus, orientation of head-regenerating tissues affected the phenotypic distribution more than increasing agarose stiffness.

To test whether soft compression was essential for phenotype generation, we compressed the head-regenerating tissues between stiffer plastic microfluidic channels of fixed thickness. At 200-μm confinement, only dead tissues were obtained, and at 400 μm, no phenotypic changes were observed (fig. S1, F and G). Moreover, compressing head-regenerating tissues between two 1% agarose slabs increased the penetrance of phenotypes while reducing the proportion of deaths (fig. S1H). We therefore concluded that soft compression was essential, probably applying sufficient constraints to cause biaxial phenotype, still allowing for contractions essential for regeneration (*7*, *8*). Our results show that soft compression of foot tissues during regeneration of the head can trigger head duplication, leading to viable bicephalous animals.

### Regenerating tissues form two heads and two aster defects during compression

We further investigated the reasons for such a marked change in morphogenetic outcome. Given the correlation between the position of actin topological defects and site of new head regeneration (*10*), we wondered how the actin nematic order was modified during compression. 3D two-photon imaging of biaxial animals (12 dpd) showed that ectodermal actin retains its long-range nematic order but that additional defects were present at the mouth position and in between the heads (fig. S2A). We wondered when these additional defects appeared under compression.

**Fig. 2.**
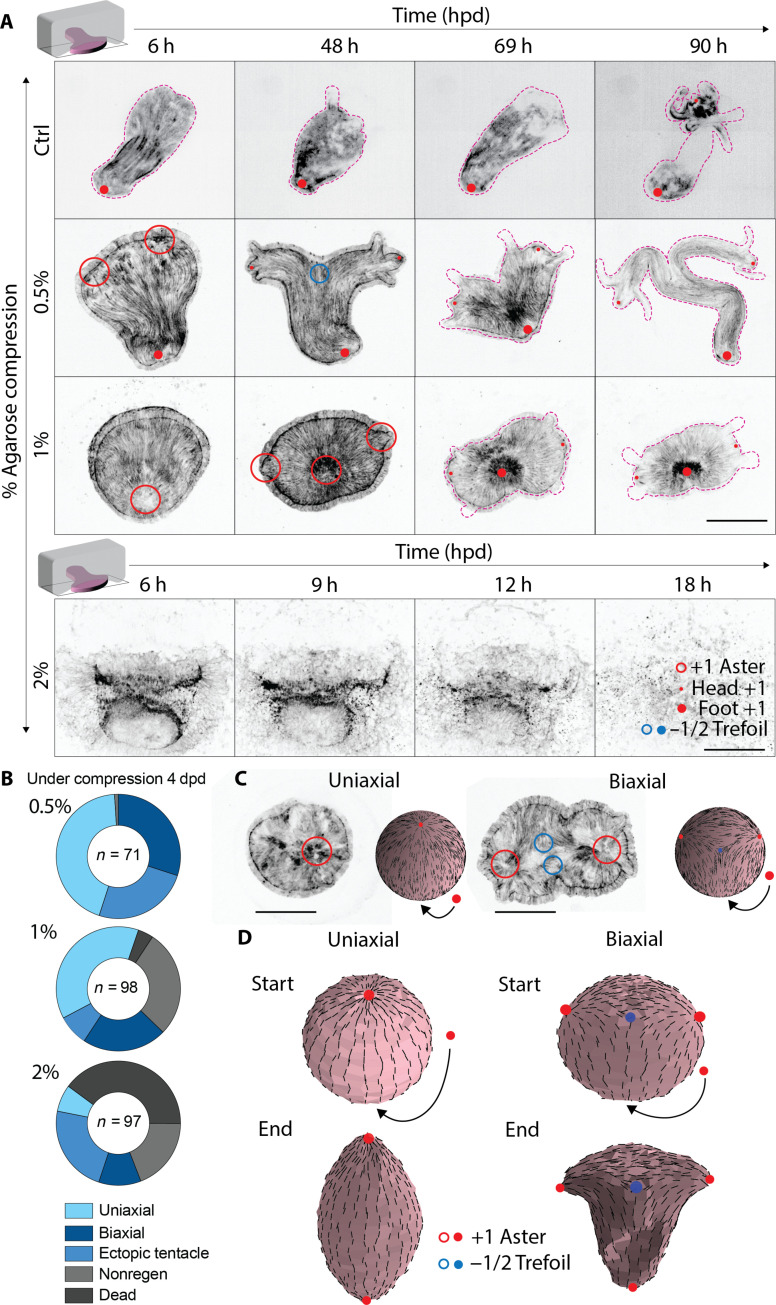
Regenerating tissues form two heads and two aster defects during compression. (**A**) Montages of live spinning disk microscopy movies (maximum intensity *z* projections) of GFP-Ecto-LifeAct head-regenerating tissues for different % of agarose compression. Control is 0.5% agarose-embedded tissues (movies S2 to S5). Pink dashed lines correspond to tissue contours. Red rings on the images correspond to +1 aster defects facing the field of view, and red dots are used for +1 aster defects observed orthogonally to the field of view. Blue ring corresponds to −1/2 trefoil defect facing the field of view. Scale bars, 100 μm. h, hours; hpd, hours post-dissection. (**B**) Ring plot showing the phenotype distribution at 4 dpd under different % of agarose compression. (**C**) Spinning disk images of head-regenerating tissues and adjacent are corresponding simulation initial states of simulation. Red rings and dots correspond to +1 aster defects, whereas blue rings and dots correspond to −1/2 trefoil defects. (**D**) Start and end states of simulations of deformations of active nematic spheres corresponding to uniaxial and biaxial conditions. Left column corresponds to uniaxial, with two aster defects (red dot; the second—symmetrical—defect is not visible and is depicted by the curved arrows pointing out of plane); right column, biaxial with three aster defects (red dots; the second—symmetrical—defect is not visible and is depicted by the curved arrows pointing out of plane) and compensating −1/2 trefoil defects (blue dots). Scale bars, 100 μm.

To visualize actin order during regeneration, head-regenerating tissues were prepared from Lifeact–green fluorescent protein (GFP)-expressing animals (*11*) and imaged under compression by live spinning disk confocal microscopy. Head-regenerating tissues inherited the actin nematic order, with a single topological defect on the basal disc and longitudinal fibers expanding toward the regenerating wound (Fig. 2A, Ctrl, and movie S1) (*10*). Upon compression, the wound edge was notably flattened out and regenerated two heads at the tissue extremities (Fig. 2A, 0.5% & 1%) in 30% of the 0.5% AC animals within 4 dpd (Fig. 2B and movie S4). In animals that successfully regenerated, actin order was retained throughout regeneration, confirming that long-range actin order was mostly kept during compression (Fig. 2A).

During regeneration under 0.5% AC, proportions of each phenotype were similar than observed previously in the fully regenerated animals (Fig. 2B, 0.5%). In less than 1% of the cases, tissues did not regenerate but remained viable until the end of compression (4 dpd). The nonregenerative fraction significantly increased in 1% AC (28%) as higher stiffness may slow down the tissues’ contractions (movie S5), delaying regeneration, and death became significant (Fig. 2B, 1%). For 2% AC, 40% of the head-regenerating tissues underwent death, whereas uniaxial tissues became minor (Fig. 2B, 2%). Thus, the mechanically induced phenotypes appeared during compression, and increasing agarose stiffness increased their prevalence and death. Therefore, phenotypes visible in the released animals (Fig. 1, D and E) appeared during and because of compression (Fig. 2, A and B, and movies S4 and S5).

In 1 and 2% AC, some of the head-regenerating tissues regenerating two heads were orally oriented, with the mouth/wound side facing the glass. In this case, two +1 aster defects were clearly visualized whereas the two heads emerged (Fig. 2C), consistent with the reported correlation between +1 aster defect position and head regeneration (*10*). We concluded from these results that the regeneration of two heads correlated with the emergence of two aster defects under compression. On the basis of our previous findings that integer topological defects can organize cellular stresses that shape tissues, we imagined that the tissue shape required for regenerating the head could be generated by the actin stress field around the defects (*14*). We further envisioned that a second defect could create a tissue shape adapted to a second head.

To test these hypotheses, we undertook a theoretical approach. We approximate the head-regenerating tissue as a thin elastic nematic material in which active stress is generated parallel to the nematic orientation. We approximate the initial shape of the regenerating *Hydra* as a sphere, and the actin supracellular organization is described by the nematic orientation field on its surface. We assume that all nematic orientation fields feature an aster at the pole corresponding to the foot. The remaining orientation field is broadly inferred from experimental images. For uniaxial regeneration, the actin supracellular organization features a single aster on the mouth pole (Fig. 2C and fig. S3A). At the end of uniaxial simulations, the active elastic adopts an elongated shape similar to a single-headed *Hydra*, with defects at the tips (Fig. 2D, uniaxial, and movie S6). This deformation is explained by the fact that, in a thin elastic active nematic shell, aster defects are able to generate dome-shaped protrusions by organizing active stress (*19*, *20*). During biaxial regeneration of *Hydra*, we observed a third aster associated with the additional head (Fig. 2C and fig. S4A), which we included in the initial nematic orientation field; this required an additional pair of negative defects to preserve the total topological charge (Fig. 2D, biaxial). During biaxial simulations, the spherical surface undergoes a splitting of the central axis, resulting in a biaxial shape (Fig. 2D, biaxial, and movie S7). Thus, our simulations show that the shape of a second head can emerge from an additional aster in the orientation field of the supracellular actin. Our results show that each head regeneration correlates with an aster defect of the actin supracellular organization on the head-regenerating tissue, supporting the notion that an actin defect is required to shape a head.

### Defect position more than mechanical properties of tissue governs shape generation

As the possible role of defect would be to organize active stresses to shape tissues, the final shape should depend on tissue’s mechanical parameters. In the simulations, the thickness of the active nematic shell controls its rigidity: Very thin materials are relatively easier to bend. The size of the defects governs how far the loss of order propagates. At distances much larger than the defect size, the order parameter *S* = 1, i.e., perfect order, and at the core of the defect, *S* = 0. Thus, defect size controls the global intensity of the nematic order and thus how much active stress is generated (fig. S3B).

By modifying these two key simulation parameters, (i) the thickness of the nematic shell (*t*) and (ii) the size of the defect (i.e., defect core radius, ε), we show that the degree of shape change varies with these parameters. As expected, increasing thickness and thus rigidity reduces the deformation, whereas reducing ε, increasing order and active stresses, increases deformation (fig. S3C). However, over a fivefold change for thickness and a 10-fold change for defect size, the global shape that results from active stress is the same (fig. S3C), essentially imposed by the position of defects (fig. S3A). Similar results were obtained with two-headed shapes (fig. S4). Thus, our simulations show that, although rigidity and strength of active stresses modify the shape of the regenerating tissue, the position of topological defects has a more important impact on setting the final shape (figs. S3 and S4).

To relate this to experimental data, we measured the orientation field of actin in a typical Hydra using structure factor method (OrientationJ plugin for ImageJ). We then coursed this orientation field to estimate the local order parameter. In all our images, the order parameter is close to the maximum value = 1 outside of regions of high curvature and topological defects, corresponding to the case of very small defect size in simulations. About rigidity, a recent study measured the Young’s modulus of the *Hydra* tissue (*E* ~ 440 Pa) (*21*). As the Young’s modulus of epithelial monolayers is in the range of 20,000 Pa (*22*), the *Hydra* tissue is 50 times softer than epithelial cells, which are easily deformed by internal stresses (*23*). Therefore, *Hydra* correspond to the simulation case where order is maximal and rigidity is the lowest, showing the highest deformability.

To appreciate the relative importance of the placement of topological defects on the tissue final shape, we focused on the two-headed *Hydra* as it has more degrees of freedom. We show that, even when the positions of the foot and head defects are fixed, a range of *Hydra* shapes are predicted by only adjusting the positions of the negative defects (fig. S5, A and B). This corresponds to the variety of multiheaded phenotypes we observe experimentally (fig. S2B), supporting the notion that placements of defects on the surface of the tissue controlled the shape.

Simulations show that the final shape of the *Hydra* is highly robust to changes in the material parameters of the model but greatly affected by the locations of the topological defects. We therefore wondered how the absence of topological defects would change the *Hydra*’s regeneration.

### Defectless toroids fail to regenerate

**Fig. 3.**
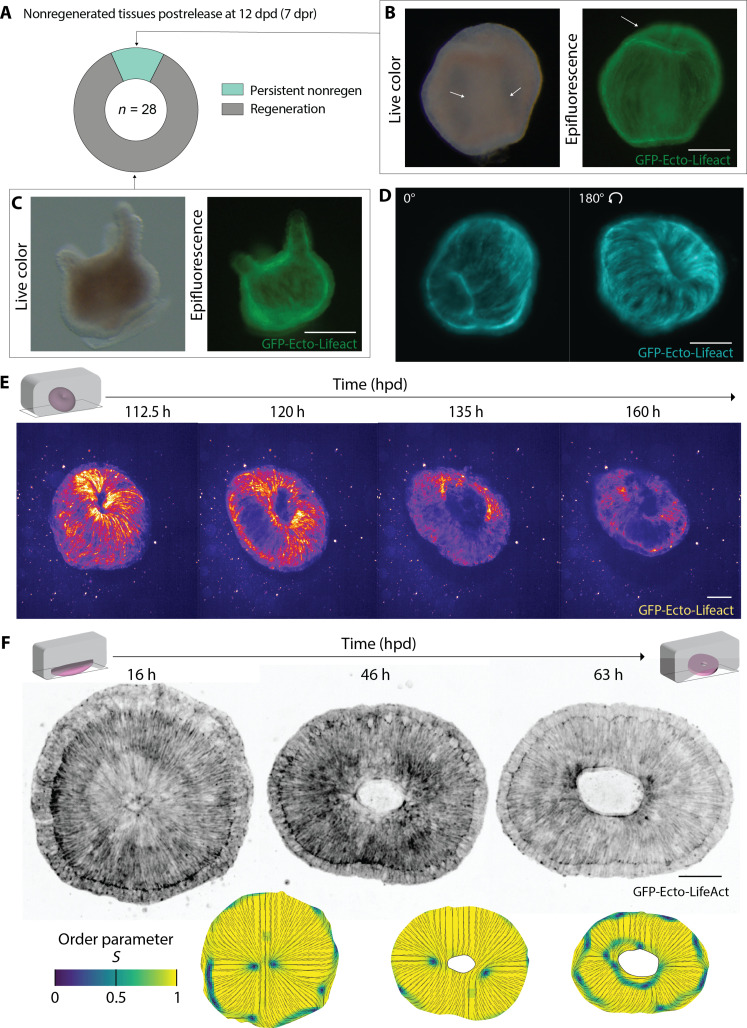
Defectless toroids fail to regenerate. (**A**) Ring plot showing the fate of nonregenerated tissues after 4 dpd compression and screened at 12 dpd [7 days postrelease (dpr)]. (**B**) Left: live color image of persistent nonregenerative tissues 12 hours after compression release; white arrows show tubular tissue thickening. Right: corresponding epifluorescence image of the tissue expressing GFP-Ecto-Lifeact displaying a tissue fold at the top (white arrow). Scale bars, 500 μm. (**C**) Left: live color image of regenerated tissues 12 hours after compression release (nonregenerated after 4 dpd compression) displaying tentacles. Right: corresponding epifluorescence image of the tissue expressing GFP-Ecto-Lifeact. Scale bars, 200 μm. (**D**) Maximum intensity projections of a light sheet microscopy stack of GFP-Ecto-Lifeact–expressing *Hydra* toroid. Left: top view. Right: bottom view. Scale bar, 100 μm. (**E**) Spinning disk microscopy time lapse (maximum intensity *z* projections) of GFP-Ecto-LifeAct–expressing *Hydra* toroid embedded in 0.5% agarose. Fire look-up table (LUT) was applied to appreciate three dimensionality. Scale bar, 100 μm. (**F**) Top row: spinning disk microscopy time lapse (maximum intensity *z* projections) of GFP-Ecto-Lifeact head-regenerating tissue forming a toroid. Scale bar, 100 μm. Bottom row: color map legend corresponding to order parameter *S*. Images displaying orientation images of actin fibers in black and orientation order map displayed in color where yellow corresponds to perfectly ordered regions and dark blue corresponds to loss of order highlighting sites of topological defects. Note that orientation of filaments perpendicular to the tissue edges is sometimes detected by the algorithms as lines of defects as the edge is wrongly detected as a rapid change of orientation. These lines follow the tissue boundaries and do not correspond to an actual change of orientation. h, hours; hpd, hours post-dissection.

To test further the requirement of actin defects, we next turned our attention to head-regenerating tissues that failed to regenerate under compression, previously referred to as nonregenerative tissues. These may just be delayed in regeneration or may lack features essential to regeneration. To test whether compression had simply slowed down regeneration, we determined the ability of 1% AC nonregenerative tissues to regenerate after compression release. These tissues were released from compression at 5 dpd, and a large proportion of these nonregenerative tissues did regenerate as screened at 12 dpd (>80%, Fig. 3A). The remaining 20% (~5% of the initial pool) were unable to regenerate even after release. We defined them as persistent nonregenerative tissues (Fig. 3B). Bright-field imaging 12 hours after release showed the presence of a longitudinal tube-like thickening at the center of the persistent nonregenerative tissues (Fig. 3B and fig. S6A). Epifluorescence imaging revealed the presence of a tissue fold forming a tunnel through the tissue resulting in the topology of a torus (Fig. 3B). By comparison at 12 hours after release, tissues with a spherical topology had already regenerated primitive tentacles (Fig. 3C). 360° light sheet microscopy confirmed the toroidal topology of the persistent nonregenerative tissues, hereafter called toroids. These images also showed that the actin orientation field displayed rotational symmetry, thus featuring no defects (Fig. 3D and fig. S6B). As its Euler characteristic equals zero, the torus is the only topology supporting a fully ordered actin superstructure, with no defects. The lack of defects in toroids could therefore be associated with their inability to regenerate.

To confirm the lack of regeneration after release from compression, toroids were imaged live. The toroids retained their shape over 60 hours after release and did not break symmetry in their actin order nor regenerate (Fig. 3E and movie S8). The toroids eventually disintegrated before 12 dpd, explaining why only dead or regenerated animals were observed at 12 dpd (Fig. 1C). In conclusion, head-regenerating tissues that acquired the topology of a torus, with no topological defects in the actin superstructure, could not regenerate.

We then studied how this unique topology change could occur under compression. In aboral and oral orientations, head-regenerating tissues occasionally underwent tissue tear right at the center of the actin aster, suggesting a local increase in stress (Fig. 3F). A mirror tear occurred at the antipole of the head-regenerating tissue. The tissue tears during contractions, which are oriented radially around the defect (movie S9). Wound healing occurs along the body axis fusing the two tears, annihilating the two aster defects and giving rise to a defectless toroid (Fig. 3F) (*24*). The emergence of tissue tearing at defect locations can lead to a change in topology. A recent study (*25*) has also correlated tissue fractures with such mechanical stress at defect locations.

**Fig. 4.**
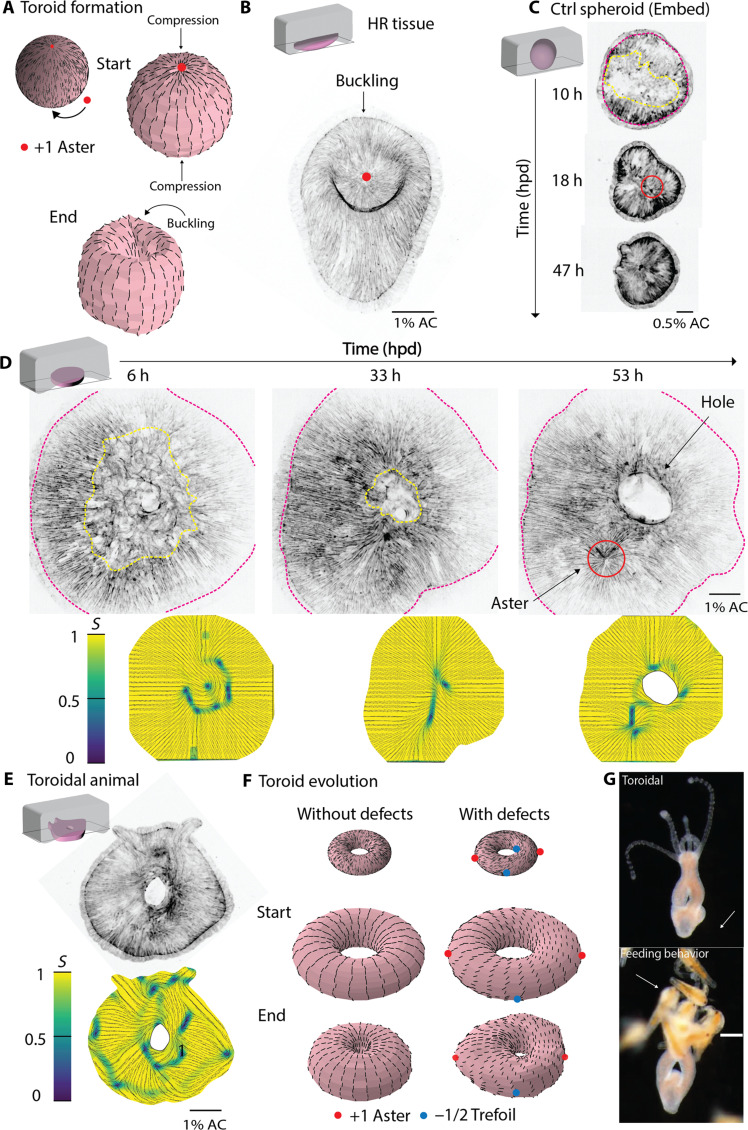
Actin topological defects shape heads in *Hydra* toroids. (**A**) Simulation of toroid formation under compression. Top: initial order of an active nematic sphere. Middle: active nematic sphere shape at simulation start, with arrows indicating compression orientation. Bottom: active nematic sphere shape at simulation end, with +1 aster defects shown as red dots. (**B**) Head-regenerating tissue under compression, buckling at a +1 aster defect at the aboral end. Red dots indicate +1 aster defects. (**C**) Regenerating spheroid tissue embedded in 0.5% agarose (control). Pink dashed line shows tissue contours; the yellow line marks areas with loss of actin order. The red ring shows +1 aster defects. (**D**) Regenerating spheroid tissue under 1% AC forming a +1 aster defect. Pink dashed line shows tissue contours; yellow line indicates actin disorganization. Red ring shows +1 aster defect. Bottom: color map of order parameter *S*; actin orientation in black and order (yellow, ordered regions; blue, disordered regions corresponding to topological defects). (**E**) Toroidal tissue under 1% AC regenerating a head at 84 hpd. Bottom: order parameter image. (**F**) Initial states of nematic spheres and toroid simulations with/without defects. Red dots indicate +1 aster defects; blue dots, −1/2 trefoil defects. Scale bars, 100 μm. (**G**) Bright-field images of a toroidal animal (top) and its feeding behavior (bottom). Scale bar, 200 μm. h, hours; hpd, hours post-dissection.

As mentioned previously, aster defects deform thin active nematic elastic materials into a protrusion, and the compression against the defect could invert the protrusion (*19*). As a consequence, the two ends of nonregenerative tissue would buckle inward under axial compression, which only occurs for oral or aboral orientations. To test this, we initialize our simulations with two asters placed at opposite poles and compression along the same axis. Simulations show that compression inverts the protrusions that result from aster defects (Fig. 4A and movie S10). In our experiments, we observed the same inversion of protrusions associated with asters at foot sites under compression (Fig. 4B and fig. S6C). This simultaneous inward buckling of tissue defects aids in the fusion event required for toroid formation (Fig. 3F) while preserving the symmetry allowing for a defectless actin superstructure.

### Actin topological defects are necessary to shape head regeneration in *Hydra* toroids

Changes in topology are noncontinuous changes in a shape: they require tearing and/or fusion of surfaces and are only witnessed during drastic stages of morphogenesis such as gastrulation. To discriminate whether the change of topology or the lack of defects blocks regeneration, we aimed to generate toroids with topological defects and test whether they would regenerate. Previous works (*1*, *26*) showed that the excised body column tissue of *Hydra* reported partial loss of supracellular actin order during initial stages of regeneration while it reshapes as a sphere, referred to as spheroid hereafter (Fig. 4C) (*26*). Spheroids have been shown to regenerate into both uniaxial and biaxial animals (fig. S7A), suggesting that spheroids can have additional defects, generating new heads. We hypothesized that, when subjected to compression, a fraction of spheroids will undergo tear and form toroidal tissues featuring additional topological defects.

Spheroids under compression were imaged by live spinning disk confocal microscopy. Large areas where actin lacked nematic order were visible (Fig. 4, C and D). Initially, no topological defects were seen, but 16 hpd, the actin superstructure emerged and topological defects appeared (Fig. 4D, time 33 to 53 hpd, and movie S11). As expected, certain tissues underwent tear and changed topology before global actin order was established (Fig. 4D). This is opposed to the topological change of toroids reported above, which occurs with preserved actin order. Furthermore, aster defects were clearly visible in the actin superstructure of spheroids when the tissue was still under compression undergoing wound healing (Fig. 4D, time 53 hpd, and movie S12). At the position of these defects, head and foot features appeared, showing that toroidal tissues with defects are able to regenerate (Fig. 4E).

Among the regeneration features that appeared on toroidal tissues with defects were protuberances on their contour, reminiscent of dome shapes coupled to aster defects (Fig. 4, D and E) (*19*). To test whether these protuberances could be created by stress gradients linked to defects, we simulated toroidal surfaces with and without defects (Fig. 4F). Simulations of toroids with no defects fail to break symmetry and remains perfectly toroidal (movie S13), whereas simulations featuring defects break symmetry and form protuberances colocalizing with defects (movie S14).

To test whether toroids with defects would further regenerate into viable animals, we released them from compression at 5 dpd and observed that they were able to recover and thrive. After compression, we observed a multitude of phenotypes including toroidal animals with a head and foot (Fig. 4F) and new phenotypes such as bipedal animals (fig. S7A). The induction of tissue tear and consequent wound healing depended on the stiffness of agarose used for compression as toroidal animals were only generated using 1% AC (fig. S7, A and C). These animals have fully functional head and foot, able to trap *Artemia* (movie S15), while having a hole that bifurcates their gastrovascular cavity (Fig. 4G). Overall, *Hydra* toroidal tissues with actin defects regenerate live toroidal animals with a head and a foot, conserving their unique topology, showing that actin topological defects are necessary for forming a new head and establishing body axis.

## DISCUSSION

Overall, these results strongly support a necessary role of actin integer topological defects in the regeneration of *Hydra*’s head. Simulations based on active nematic theory support that these actin defects establish the stress field required for positively curved surfaces shaping the new head. These simulations are supported by the observations that tearing and buckling occur predominantly at the defects, as expected from the stress field calculated from the theory. How the mechanical role of actin topological defects is coupled to the Wnt organizer remains to be explored, but our experiments show that actin topological defects are as essential as Wnt organizers in inducing the full program of head regeneration. Recent findings propose that stretching of cells associated with larger stresses at the defects may be coupled to Wnt production through mechanosensing signaling (*27*–*31*), in line with past findings suggesting a tight mechanochemical coupling in the animal (*7*, *8*, *21*).

The common topology of developing and regenerating tissues is equivalent to the one of a sphere, with a total charge of +2. Our work shows that topology is an important constraint that participates in the establishment of the body axis and thus the body plan. Topology thus may have participated in the selection of common features of body plans through evolution. It is also notable that embryos change their topology at critical steps during their development: not only during gastrulation but also during somitogenesis or neurulation. Thus, genetically controlled changes in the embryo topology may participate in establishing the final body plan. Our work opens important perspectives for understanding not only how topological changes are required for morphogenesis but also on how topology could be used to create new body plans.

## MATERIALS AND METHODS

### Animal culture

All of the experiments were performed using transgenic *Hydra vulgaris* (strain Basel) expressing Lifeact-GFP in the ectodermal cells provided by B. Hobmayer from the University of Innsbruck, Austria (*11*).

Cultures were maintained in a *Hydra* medium (HM) [1 mM NaCl, 1 mM CaCl_2_, 0.1 mM KCl, 0.1 mM MgSO_4_, and 1 mM Tris (pH 7.6)] at 18°C. Animals were fed two to three times per week with freshly hatched *Artemia nauplii* and starved for 24 hours before any experiment. Nonbudding animals that had fed were chosen at random from the dish.

### Sample preparation

Animals approximately greater than 5 mm long were transversely sectioned into three equal parts. The head tissue was discarded. The body column was left to heal for 6 hours to form sealed spheroids. The bottom third containing the foot was left to heal for 6 hours and used as the head-regenerating tissue. A scalpel equipped with a no. 23 blade was used for dissections.

For control experiments, spheroids and head-regenerating tissues were embedded in a soft gel [0.5% low-melting-point agarose (Sigma-Aldrich) prepared in HM] (*10*). The regenerating tissues were placed in cooled down liquefied gel 6 hpd, and the gel was left to solidify. The tissues naturally settled down to the glass bottom due to their density, which made it easier to image them.

### Compression experiments

Twenty milliliters of liquefied agarose (0.5, 1, or 2%) was poured in a 10-ml petri dish to obtain a constant height for the agarose slabs. The agarose was allowed to solidify with closed lids to prevent evaporation. Once cooled, regenerating tissues were placed on the solidified agarose. With the tissue at the center, an approximate square cut (5 mm by 5 mm) is made in the agarose with a no. 23 scalpel. Then, the agarose slab containing the tissue on its surface is gently excised out of the petri dish with the scalpel and flipped over tissue side first on a 35-mm glass-bottom MatTek dish, trapping the regenerating tissues between the glass bottom and the excised agarose slab. Extreme precision is required during this step to avoid tissue shear and subsequent disintegration. After placing roughly four to five agarose compressed tissues in each MatTek dish, 3 ml of liquefied agarose is poured on top to avoid evaporation and preventing tissues wiggling their way out of the compression. After the agarose has been solidified, 2 ml of HM is added to each petri dish and lids were placed to further prevent evaporation during the course of 4 days of regeneration at 18°C.

For agarose/agarose compression experiments, the MatTek dish is coated with 1 ml of 1% agarose prior to compressing tissues with 1% agarose slabs.

### Compression release

At 5 dpd, the supernatant HM is removed from each dish. Cuts (5 mm by 5 mm) are made in agarose around the tissues in the center, located at the bottom. The HM is flushed into the cuts, which enables the agarose slab to float and with it the compressed tissue at the bottom. This enables us to minimize any shear faced by the tissue during compression release. The suspended tissue/regenerated animal is collected with a glass Pasteur pipette and moved to a new well with fresh HM.

For experiments where phenotypic screening was performed, all the tissues were labeled on the MatTek dish with corresponding numbers and, when released, placed in labeled wells. This animal labeling enabled us to identify the correlation between tissue orientation and what they regenerated into when their phenotype was screened upon compression release.

### Microfluidic channel confinement

Ibidi sticky slides (200 and 400 μm) were used for channel confinement experiments. The tissue was placed in the sticky channel, and excess water was removed and then sealed with a glass coverslip. The HM was then flushed into the wells gently and replenished every day to compensate for evaporation loss. The tissues were confined for 4 days before images were taken to screen for phenotype.

### Microscopy

All time-lapse imaging was performed with an inverted microscope Nikon Ti-E installed in a room where the temperature was maintained at 20°C. The microscope was also equipped with an automated stage and a Yokogawa CSU-W1 spinning disk unit. Image acquisition was performed with an Andor Zyla 4.2 Plus camera, operated with the SlideBook software. Fluorescence 4D time-lapse imaging was performed to capture actin dynamics in regenerating tissues either embedded or under agarose slab compression using a 10x [numerical aperture (NA) 0.30] objective. For all experiments under compression, we acquired two images/hour for 84 hours. All movies are maximum intensity *z* projections of 42 *z*-stacks each spaced 4 μm apart.

Light sheet microscopy was performed on a Miltenyi Biotec Ultramicroscope Blaze Light Sheet equipped with a 4.2-megapixel scientific complementary metal-oxide semiconductor (sCMOS) camera. A 4x (NA 0.35) objective was used with 2.5x zoom. The toroid was embedded in an agarose cube with HM buffer and imaged in the light sheet. The agarose cube was manually rotated to obtain different angles of the tissue. Stacks (4 μm in thickness) were taken. A total of 184 *z*-stacks were taken, and the maximum intensity was *z* projected to compile final images.

An upright multiphoton confocal microscope (Leica SP8DIVE FALCON) equipped with HyD detectors was used to obtain the 3D images of bicephalous animals. Live imaging was possible, and the ability to penetrate the tissue was achieved by using z-compensation mode where the laser intensity was increased with increasing depth into the tissue. Tunable multiphoton laser was generated at 820 nm to excite the GFP-tagged Lifeact at 488 nm. A 25x (NA 0.95) IRAPO (maximum transmission of infrared and visible and minimal axial shift up to 1300 nm) water immersion objective was used to image the samples. *z*-stacks (4 μm) were used to image the samples in 3D.

### Whole-mount in situ hybridization

*Hydra* at 12 dpd (7dpr) were relaxed in 2% urethane/HM for 1 min, fixed in 4% paraformaldehyde (PFA) prepared in HM (pH 7.5) for 4 hours at room temperature (RT), and stored in MeOH at −20°C for at least 1 day. Samples were rehydrated through a series of ethanol and PBSTw (phosphate-buffered saline and 0.1% Tween) washes (75, 50, and 25%) for 5 min each, washed 3× with PBSTw for 5 min, digested with Proteinase K (10 μg/ml; Roche) in 0.1% SDS and PBSTw for 10 min, stopped by adding glycine (4 mg/ml), and incubated for 10 min. Samples were washed 2× in PBSTw for 5 min, treated with 0.1 M triethanolamine (TEA) for 2 × 5 min, and incubated for 5 min after adding 0.25% acetic anhydride (v/v) and 5 min after adding again 0.25% acetic anhydride (final concentration, 0.5% v/v). Samples were then washed in PBSTw 2 × 5 min, postfixed in 4% formaldehyde and PBSTw for 20 min, washed in PBSTw 4 × 5 min before adding the prewarmed prehybridization buffer [PreHyb: 50% formamide, 0.1% CHAPS, 1× Denhardt’s, heparin (0.1 mg/ml), 0.1% Tween, and 5x SSC] and incubated for 2 hours at 58°C. Next, 350 μl of a hybridization buffer [PreHyb containing transfer RNA (0.2 mg/ml) and 5% dextran] containing 200 ng of digoxigenin (DIG)-labeled riboprobe was heated for 5 min at 80°C and then placed on ice for 2 min. This mix was added onto the samples and then incubated for 19 hours at 58°C. Next, the samples were rinsed 3x in prewarmed PostHyb-1 (50% formamide and 5x SSC) and successively incubated for 10 min at 58°C in PostHyb-1, PostHyb-2 (75% PostHyb-1, 25% 2x SSC, and 0.1% Tween), PostHyb-3 (50% PostHyb-1, 50% 2× SSC, and 0.1% Tween), and PostHyb-4 (25% PostHyb-1, 75% 2× SSC, and 0.1% Tween). Samples were then washed 2× 30 min in 2× SSC and 0.1% Tween, 2× 30 min in 0.2x SSC and 0.1% Tween, 2× 10 min in MAB-Buffer1 (1× MAB, 0.1% Tween), blocked in MAB-Buffer2 (20% sheep serum and MAB-Buffer1) for 1 hour, and incubated with anti-DIG-AP antibody (1:4000, Roche) in MAB-Buffer2 overnight at 4°C.

Next, the samples were washed in MAB-Buffer1 for 4 × 15 min and then in an NTMT buffer [0.1 M NaCl, 0.1 M tris-HCl (pH 9.5), and 0.1% Tween) for 5 min and lastly in NTMT and 1 mM levamisole for 2 × 5 min. The colorimetric reaction was started by adding a staining solution [0.1 mM tris-HCl (pH 9.5), 0.1 mM NaCl, 7% polyvinyl alcohol, and 1 mM levamisole) containing BCIP-NBT (bromochloroindolyl phosphate–nitro blue tetrazolium) (Roche). The background color was removed by a series of washes in EtOH/PBSTw (30%/70%, 50%/50%, 70%/30%, 100% EtOH, 70%/30%, 50%/50%, and 30%/70%) and PBSTw 2 × 10 min. Samples were postfixed for 20 min in 3.7% formaldehyde diluted in PBSTw, washed in PBSTw 3 × 10 min, and mounted with Mowiol. All steps were performed at RT unless indicated otherwise.
