# Supplementary material for: Topology changes of Hydra define actin orientation defects as organizers of morphogenesis
Source: Sci Adv. 2025 Jan 17;11(3):eadr9855. doi: 10.1126/sciadv.adr9855 (PMC11740953; doi:10.1126/sciadv.adr9855)
Supplement: Supplementary file 1 — Figs. S1 to S9 Legends for movies S1 to S15 Supplementary Text Tables S1 to S5 [file sciadv.adr9855_sm.pdf]

Science Advances
AAAS

Supplementary Materials for

Topology changes of *Hydra* define actin orientation defects as organizers of morphogenesis

Yamini Ravichandran *et al.*

Corresponding author: Aurélien Roux, aurelien.roux@unige.ch

Sci. Adv. **11**, eadr9855 (2025)
DOI: 10.1126/sciadv.adr9855

The PDF file includes:

Figs. S1 to S9
Legends for movies S1 to S15
Supplementary Text
Tables S1 to S5

Other Supplementary Material for this manuscript includes the following:

Movies S1 to S15

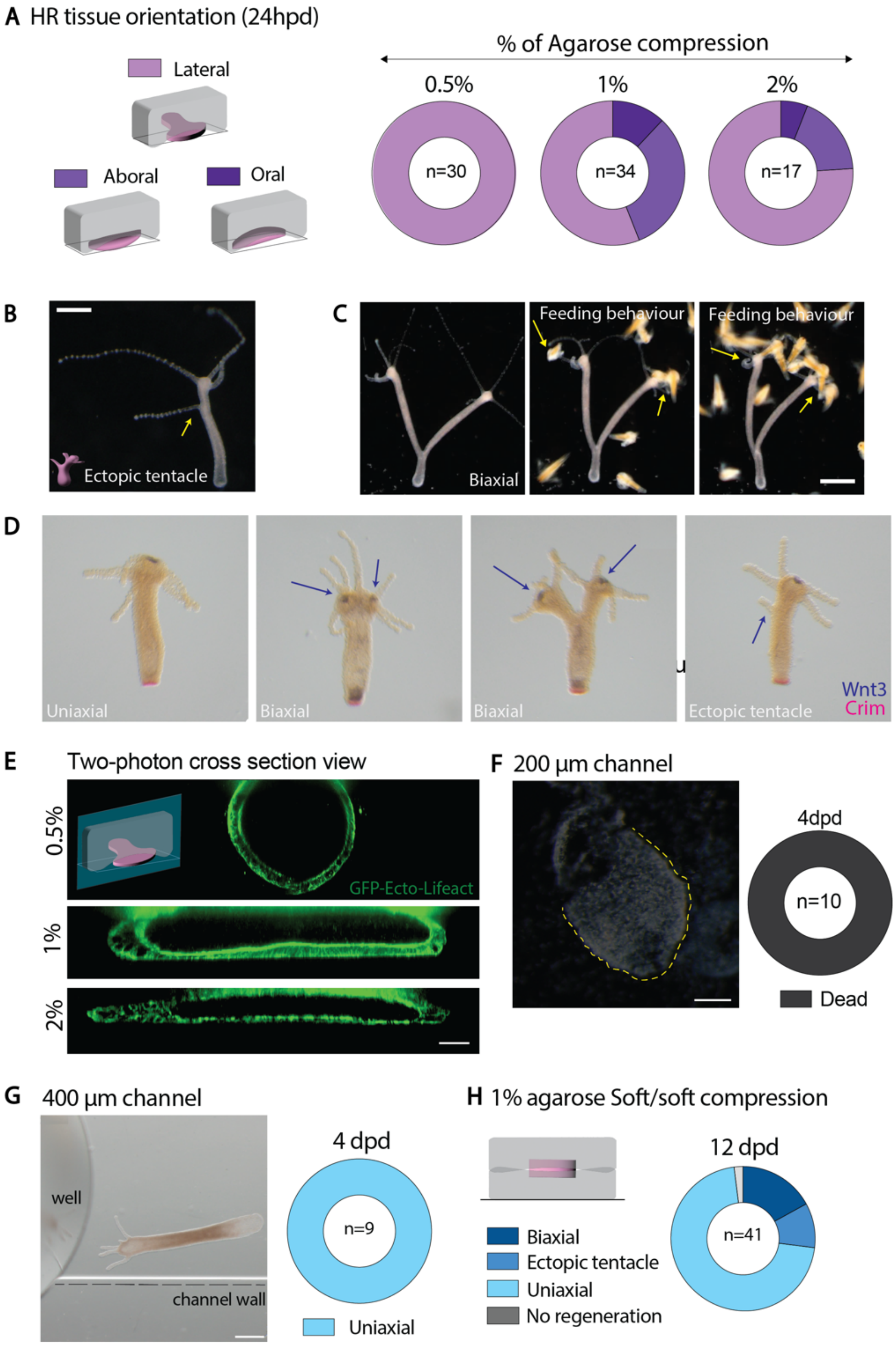
A HR tissue orientation (24hpd)
Lateral
Aboral
Oral
% of Agarose compression
0.5%
1%
2%
n=30
n=34
n=17
B
Ectopic tentacle
C
Biaxial
Feeding behaviour
Feeding behaviour
D
Uniaxial
Biaxial
Biaxial
Ectopic tentacle
Wnt3
Crim
E
Two-photon cross section view
0.5%
1%
2%
GFP-Ecto-Lifeact
F 200 µm channel
4dpd
n=10
Dead
G 400 µm channel
well
channel wall
4 dpd
n=9
Uniaxial
H 1% agarose Soft/soft compression
12 dpd
n=41
Biaxial
Ectopic tentacle
Uniaxial
No regeneration

Figure S1. (A) Ring graphs showing preferred tissue orientation at 24 hpd compressed under different percentages of agarose. **(B)** Live colour images of fully regenerated *Hydra* post-compression release at 12 dpd. **(C)** Bright-field images of one biaxial animal (right panel) and its feeding behaviour (middle and left). **(B-C)** Scale bars, 500 µm. **(D)** *In situ* hybridization experiments for Wnt3 (head marker) and Crim (foot marker). **(E)** Two-photon microscopy cross-sections of head-regenerating tissues compressed under different percentages of agarose. **(F)** Bright-field image of a head-regenerating tissue confined in a 200 µm thick microfluidic channel at 4 dpd and ring graph of regenerative outcome. **(G)** Bright-field image of a head-regenerating tissue confined in a 400 µm thick microfluidic channel at 4 dpd and ring graph of regenerative outcome. **(H)** Ring graph of regenerative outcome of head-regenerating tissues compressed under two 1% agarose slabs.

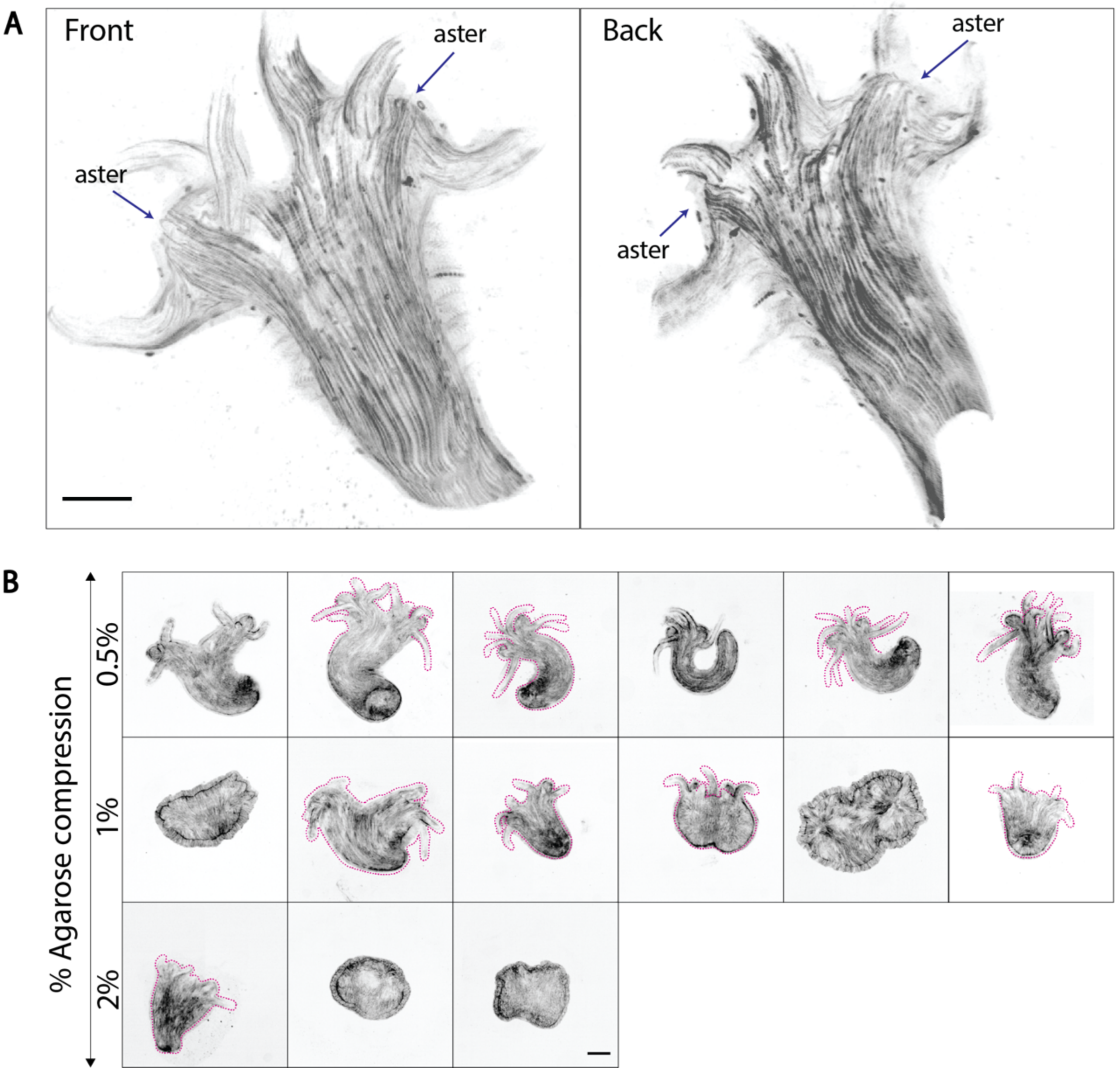

Fig. S2. (A) Two-photon microscopy (Max intensity Z-proj) of biaxial *Hydra* with front and back views of the animal with aster topological defects marked with black arrows. Scale bar, 70µm. **(B)** Live spinning disk images (Max intensity Z-proj) at 4dpd of the various head-regenerating tissues compressed under different % of agarose and displaying diversity of biaxial phenotype. Pink dashed lines correspond to tissue contours in cases where unclear. Scale bar, 100µm.

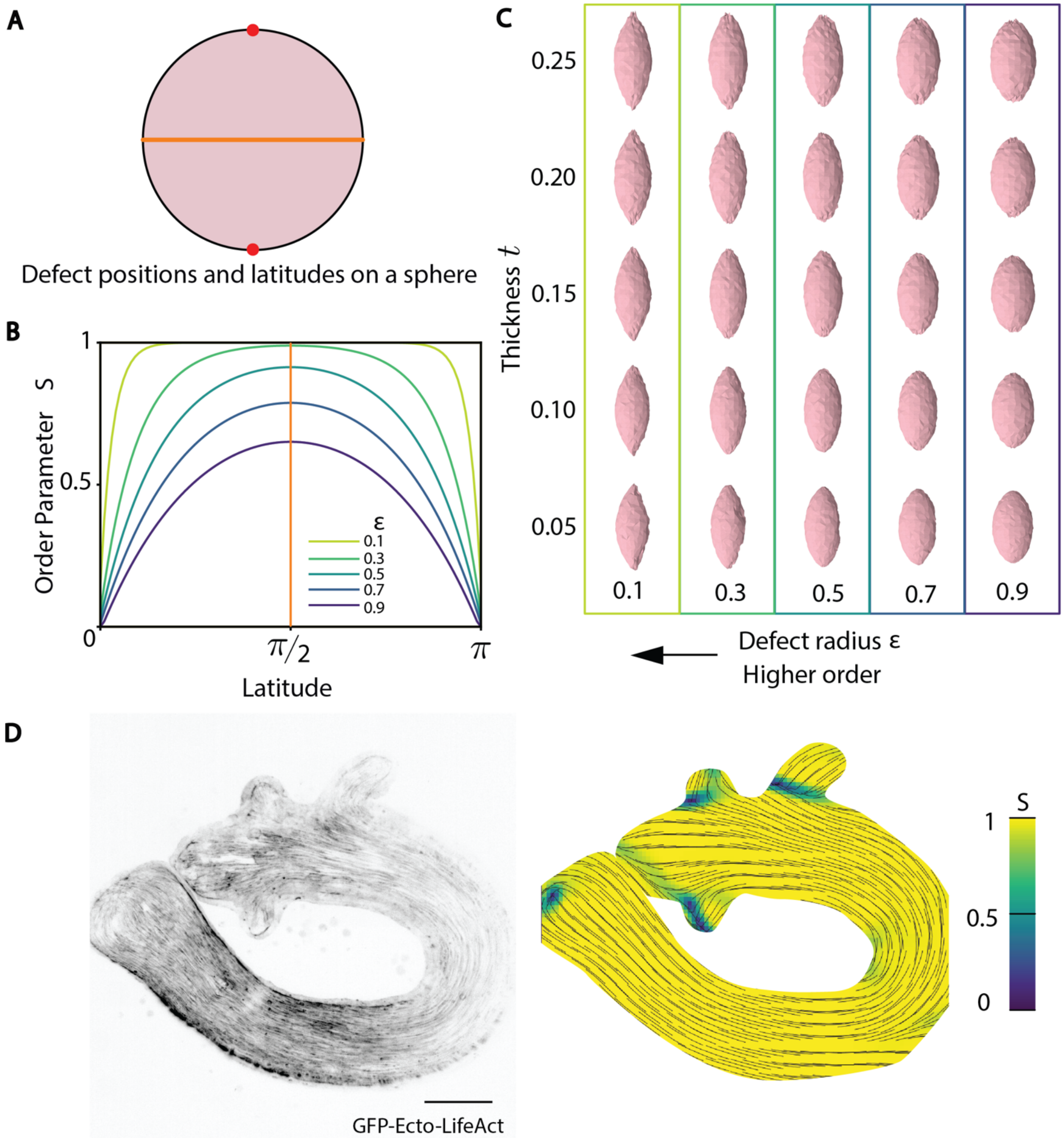

Fig. S3. Influence of mechanical parameters on single head *Hydra* shape. **(A)** Schematic showing the positions of +1 defects (red dots) at the simulation start. Also shown is the equator represented by an orange line at latitude π/2. **(B)** Initial order parameter, *S*, as a function of the latitudinal position on the sphere for various values of the defect core radius, epsilon (ε). The order parameter is always maximized at the equator and minimized at the defect positions. **(C)** Simulation outcomes for a range of defect core radii, epsilon (ε), and tissue thickness (t). All simulations give a final shape consistent with extension along the body axis provided there is sufficient order parameter, *S*, and the system is sufficiently flexible (low thickness). **(D)** Example of a typical uniaxial *Hydra* (left) and the extracted orientation field and order parameter (right). The order parameter remains close to its maximal value (1) throughout the body column of the *Hydra*.

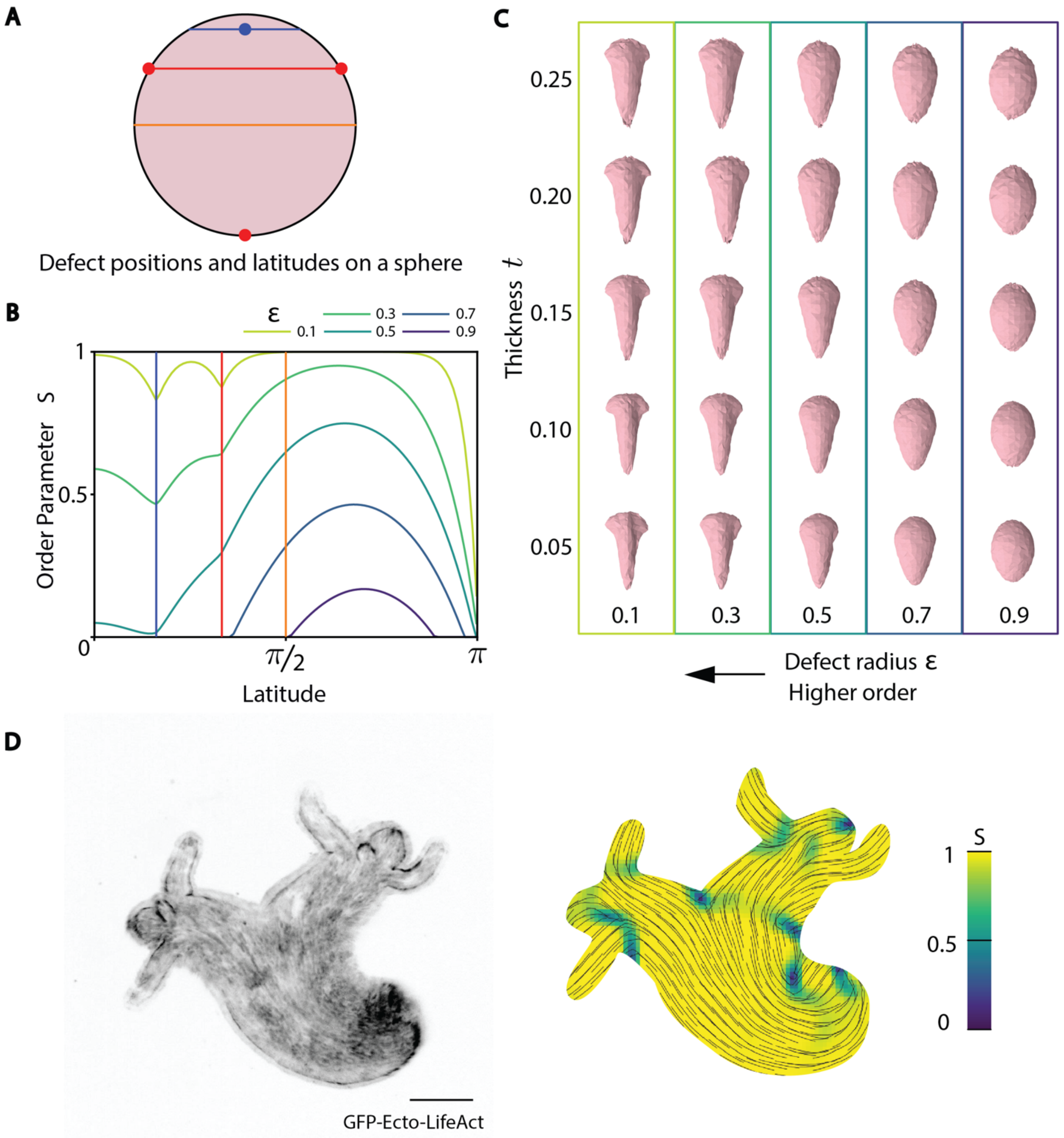

Fig S4. Influence of mechanical parameters on double head *Hydra* shape. **(A)** Schematic showing the positions of topological defects at the start of the simulation. The foot defect (red dot) is located at the south pole of the sphere, whereas the head defects (red dots) are located at latitudinal π/3 (red line). The negative defects (blue dots colocalized, see Fig S5A for 3D representation) are located at latitude π/6 (blue line). The orange line represents the equator at latitude π/2. **(B)** Average initial order parameter, *S*, as a function of the latitudinal position on the sphere for various values of the defect core radius, epsilon (ε). The average order parameter drops at the latitudes that feature defects. When the defect core radius and the areas displaying low order merge there is a total loss of orientational order far from the location of the foot (latitude π/2 to π). **(C)** Simulation outcomes for a range of defect core radii, epsilon (ε), and tissue thickness (t). For the final shape

of the simulation to have distinguishable features, the order parameter must be sufficiently high to drive the changes in shape from the initial sphere. Similarly, the tissue must be sufficiently flexible (low thickness) to realize any changes in shape. **(D)** Example of a typical biaxial *Hydra* (left) and the extracted orientation field and order parameter (right). The order parameter remains high throughout the body of the *Hydra*, compatible with the requirements of the simulations.

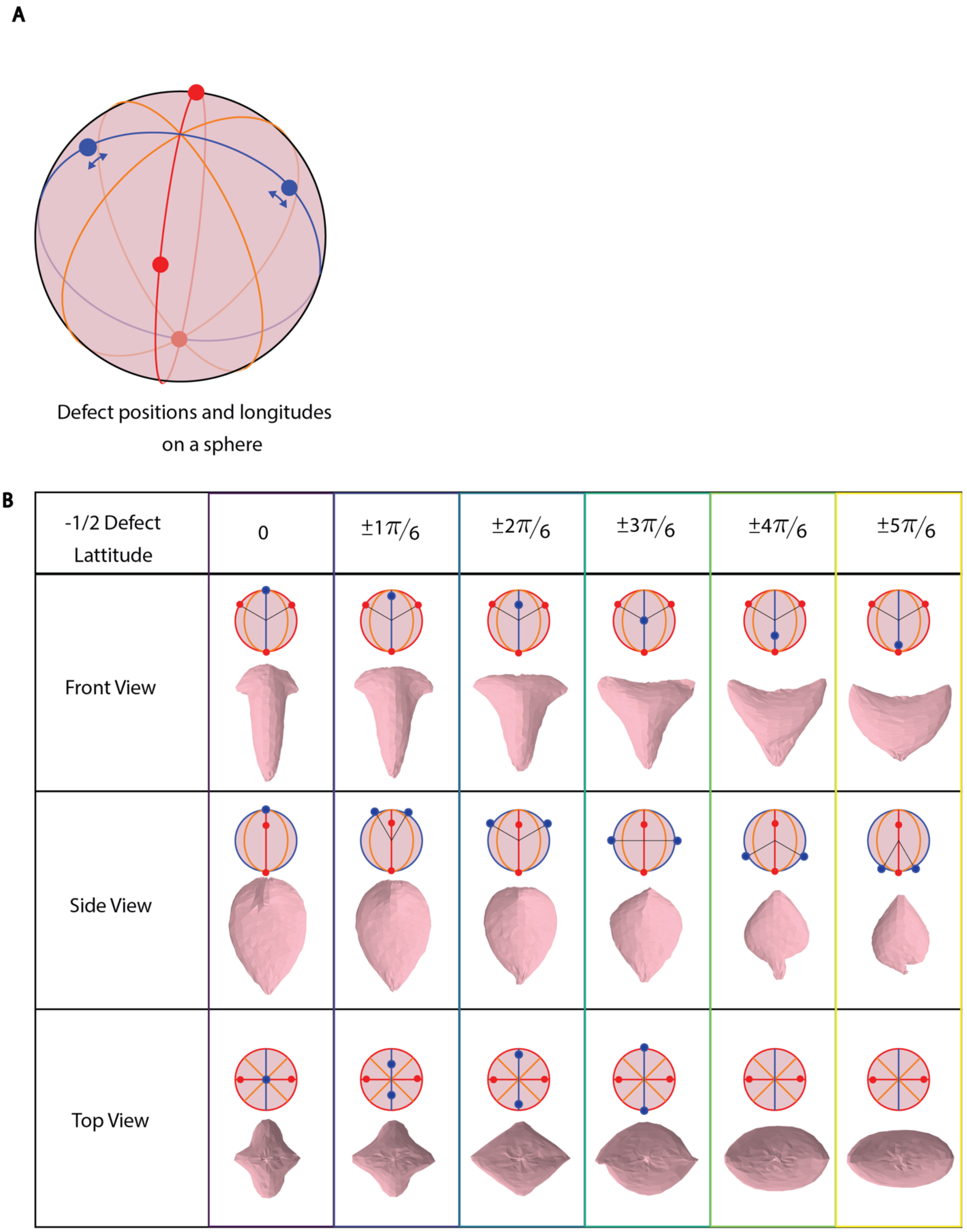

Fig S5. Influence of defect's position on double-head *Hydra* shape. **(A)** Schematic showing the initial position of defects on a sphere for a two headed *Hydra*. The positive defects (red points) dictate the foot and heads and are co-planar with the red lines of longitude. The negative defects

are coplanar with the blue lines of longitude. We assess the effect of the latitudinal position of the negative defects; the positive defects remain separated by an angle of $2\pi/3$ on the sphere. **(B)** This leads to a large range of shapes based merely on the location of the negative defects. If the defects are close to the foot, we observe biaxial animals with a shorter mid body and longer head branches. Conversely, if the negative defects are antipolar to the foot, we see a longer mid body and two short head branches. This demonstrates that the positions of the defects have far more influence on the final shape of the simulation than the exact values of the mechanical parameters.

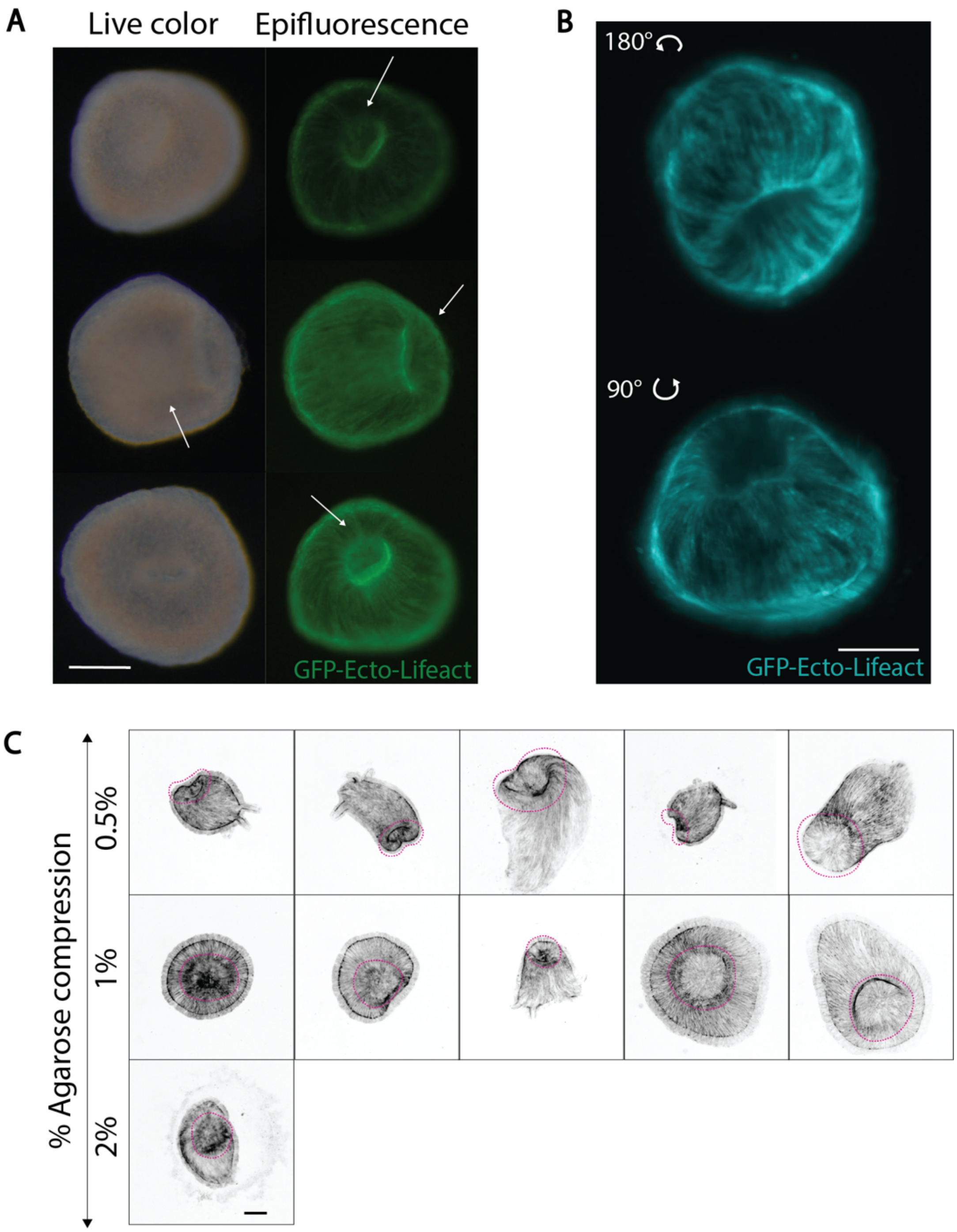

Fig. S6. (A) Left, bright-field images and corresponding right, epifluorescence images of persistent toroidal tissues with visible internal thickening and folds marked with white arrows. Scale bars, 200µm. **(B)** Light-sheet microscopy images (Max intensity Z-proj) of GFP-Ecto-Lifeact expressing toroidal tissue. Left, angled top view. Right, angled bottom view. Scale bar 100 µm. **(C)** Live spinning disk images **(**Max intensity Z-proj) at 4dpd of the various head-regenerating tissues under compression with the inward buckling invagination at the aboral end. Scale bar, 100µm.

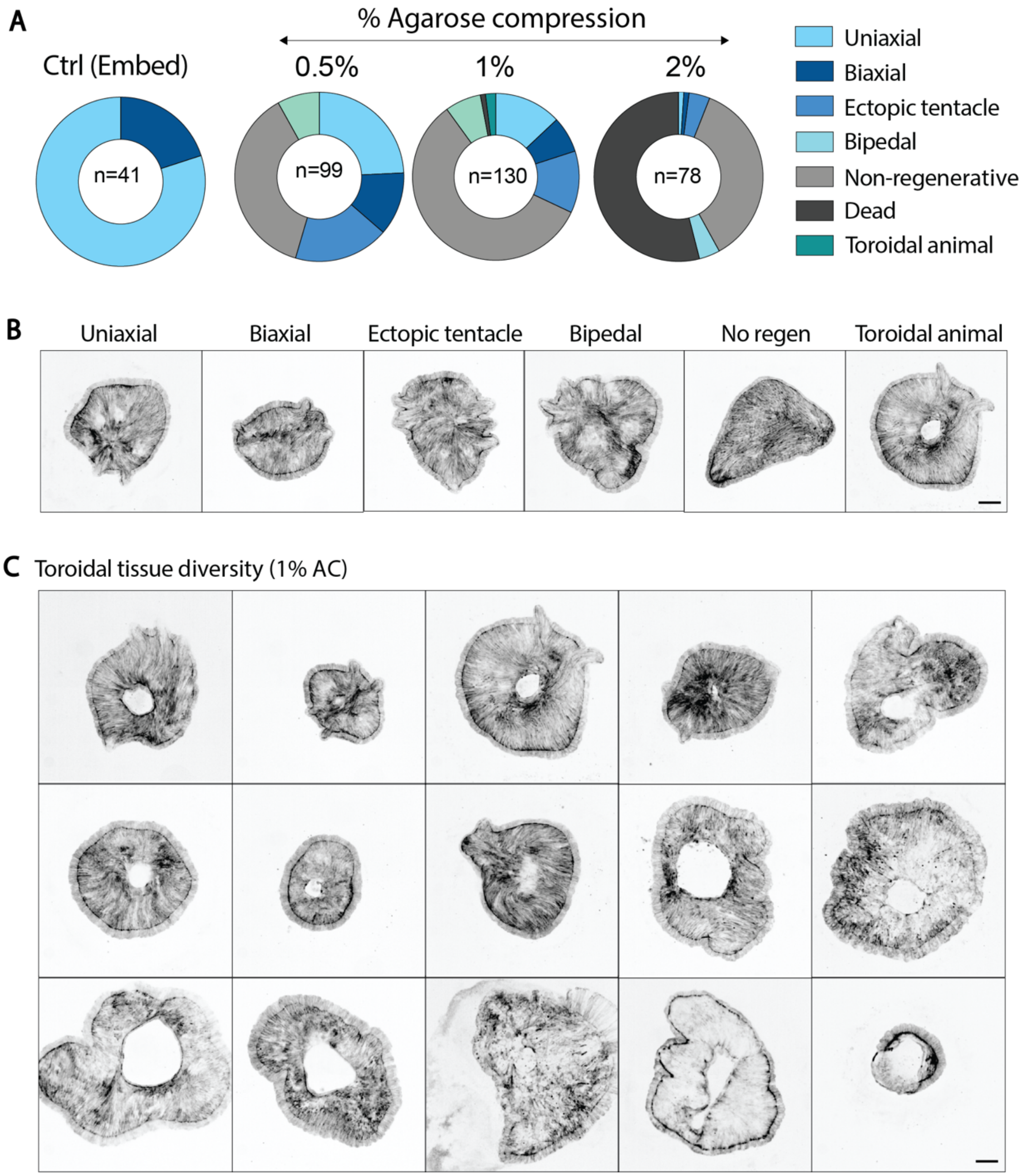

Fig S7. (A) Ring graphs showing phenotype distribution of spheroids at 4dpd compressed under different percentages of agarose. **(B)** Live spinning disk images **(**Max intensity Z-proj) at 4dpd of the various phenotypes observed with regenerating spheroids under compression. Scale bar, 100µm. **(C)** Live spinning disk images **(**Max intensity Z-proj) at 4dpd of toroidal animals obtained by compressing spheroids with 1% agarose. **(B, C)** Scale bars, 100µm.

Movie S1:

Ctrl 0.5% agarose embedded animal regenerating one head (Figure 2A) Scale bar 100 µm

Movie S2:

Biaxial *Hydra* post confinement release on Day12 post dissection displaying feeding behaviour by the ability of tentacles on both heads to trap *Artemia.*

Movie S3:

2% agarose compressed head-regenerating tissue dying by disintegration (Figure 2A) Scale bar 100 µm

Movie S4:

0.5% agarose compressed head-regenerating tissue displaying biaxial phenotype post regeneration (Figure 2A) Scale bar 100 µm

Movie S5:

1% agarose compressed head-regenerating tissue displaying biaxial phenotype post regeneration (Figure 2A) Scale bar 100 µm

Movie S6:

Simulation of uniaxial spheroid under compression (Figure 2D)

Movie S7:

Simulation of biaxial spheroid under compression (Figure 2D)

Movie S8:

Spinning disk video of toroid non regenerating post release (Figure 3E) Scale bar 100 µm

Movie S9:

Toroid formation under agarose compression (Figure 3F) Scale bar 100 µm

Movie S10:

Simulation for torus evolution and buckling mechanism (Figure 4A)

Movie S11:

Regeneration of an embedded spheroid (Figure 4C) Scale bar 100 µm

Movie S12:

Regeneration of a toroidal tissue with aster defects into toroidal animal (Figure 4D) Scale bar 100 µm

Movie S13:

Simulation of defectless toroid retains rotational symmetry (Figure 4F)

Movie S14:

Simulation of toroid with defects generating protuberances correlated with defect regions (Figure 4E)

Movie S15:

Toroidal animal feeding behaviour (Figure 4G) Scale bar 100 µm

All videos have been compressed to 10fps unless specified in movie filename and playback speed is 1x

Simulation details

I. THEORETICAL DESCRIPTION OF AN ELASTIC ACTIVE NEMATIC SOLID

To study the potential impact of the actin suprastructure on Hydra shape during regeneration, we describe its material properties in terms of an elastic active nematic solid (*19,20,28*). This is motivated by the mesoglea a gel-like elastic substance (*29*) that is sandwiched between the animal's two body layers, the (ectodermal) epidermis and (endodermal) gastrodermis. We assume that these are the mechanically most important parts of the animal. We assume that, similar to stress fibres, Hydra's actin cables in the epi- and gastrodermis generate an anisotropic active stress (*30*). A possible modulation of Hydra's material properties by genetic or biochemical regulation as well as other parts of the tissue are not part of our description.

We choose a continuum description of the material and the coarse-grained orientation of the active contractile stress is captured by an orientation field $\underline{\hat{p}}$ with $\underline{\hat{p}}^2 = 1$. It is coupled to an order parameter, $S \in [0, 1]$, accounting for the local degree of anisotropy in the active stress. Let us point out that since we assume the orientational order is nematic in character, the sign of $\underline{\hat{p}}$ is inconsequential to the mechanics of the system.

We account for the activity of the elastic material by changes of the material's reference state (*31*). In Hydra, such changes result from the internal dynamics of the actin cytoskeleton. Since we confine our analysis to the final states of Hydra regeneration, we impose a time evolution of the reference state, which interpolates between that of a sphere or a torus as the initial stress-free state and a final state incorporating contraction along the orientation field.

To analyse deformations due to changes in the reference state, we introduce an agent-based model. In this model, we partition the volume of the material into polyhedra by means of a three dimensional Voronoi tessellation. The tessellation is such that there are multiple Voronoi cells across the shortest dimensions of the material. In the simulations presented here, this corresponds to the thickness of the material and ensures resistance to bending.

The corresponding Delaunay triangulation describes a network connecting the centres of each Voronoi cell. We account for the average stress through the faces of the Voronoi cells by springs along the edges of the Delaunay network. It should be noted that the Delaunay network is not a representation of any specific structure in the active elastic material. Additionally, the Delaunay network is fixed for the duration of the simulation, thus it does not recreate the dynamics of a change in topology.

We describe changes of the reference state due to growth through time-dependent modifications of the springs' rest lengths. Explicitly, the rest length l_i of spring i at time t with initial orientation $\underline{\hat{l}}_i$ and midpoint position $\underline{r}_i$ is given by

$$l_i = \tilde{l}_i(1 + \zeta(t)) \times \sqrt{1 + \xi(t)S(\underline{r}_i) + \lambda(t)S(\underline{r}_i)[(\underline{\hat{p}} \cdot \underline{\hat{l}}_i)^2 - 0.5]}, \tag{1}$$

where $\tilde{l}_i$ is the initial rest length. The phenomenological parameters ζ, ξ and λ represent active strain coefficients. Depending on their sign, they describe relative expansion or contraction. The time-dependence of the strain coefficients reflects the evolution of the reference state. We now discuss their values in turn.

The differential anisotropic strain coefficient λ controls changes of the rest length of a connection depending on its alignment with the orientation field, hence induces anisotropic contraction or expansion. We consider linear growth up to a predetermined stall value λ^s at time t^s:

$$\lambda(t) = \begin{cases} \frac{\lambda^s t}{t^s}, & \text{if } t < t^s. \\ \lambda^s, & \text{otherwise.} \end{cases} \tag{2}$$

We choose t^s and λ such that the reference metric evolves slowly and the material remains close to mechanical equilibrium at all times. All results are presented in the long time limit ($t \gg t^s$) where the system is at a steady state.

The differential isotropic strain coefficient ξ controls changes of the rest length depending on the local order parameter, S. This then describes isotropic expansion or contraction regardless of the orientation of $\underline{\hat{p}}$ at that point. In the current study we set $\xi = 0$; an in depth discussion of the effect of ξ is available (*19*); this constrains $\lambda \in [-2, 2]$. Finally, the global strain coefficient ζ represents homogenous, isotropic expansion or contraction, thus only contributes to a global re-scaling of the system. Since we are interested in changes in morphology of the material, we set $\zeta = 0$ for all simulations.

We consider an elastic material, in which the internal dynamics of the nematic field are negligible. Consequently, the values $\underline{\hat{p}} \cdot \underline{\hat{l}}_i$ and $S(\underline{r}_i)$ are fixed at the onset of the simulation. We choose all springs to have the same spring constant, $k = 1$, and evolve the system according to an over-damped Langevin Equation. All simulations run for at least $2t^s$ and the final configurations of the model is shown in figures.

A step in our simulation consists of the following elements: First, each edge in the Delaunay triangulation is given a rest length according to Eq. (1).

The position of each point is then updated according to an over-damped Langevin equation with mobility coefficient μ, and the total stored elastic energy is given by

$$E = \sum_k \left[|\underline{r}_k(t) - \underline{r}_j(t)| - l_{kj}\right]^2. \quad (3)$$

Hence the position of point j is updated according to

$$\underline{r}_j(t+1) = \underline{r}_j(t) + \mu \sum_k \left[|\underline{r}_k(t) - \underline{r}_j(t)| - l_{kj}\right] \hat{\underline{r}}_{kj}(t), \quad (4)$$

where the points $\underline{r}_k$ are those linked to point $\underline{r}_j$ by an edge of the Delaunay triangulation, with rest length l_{kj} and $\hat{\underline{r}}_{kj}$ is a unit vector pointing from point k to point j. In all data presented $\mu = 0.05$. We simulate for $T = 10^5$ time steps with $t^s = T/2$.

This completes the description of our agent-based model.

A. Actin suprastructure in Hydra ectoderm and endoderm

In our simulations, we consider a single closed surface with finite thickness, roughly mimicking the structure of Hydra. Furthermore, we take the orientation field $\hat{\underline{p}}$ to be tangential to the surface, similar to the orientation of actin cables in Hydra. We recall that the resolution of the simulation is chosen to ensure multiple Voronoi cells over the thickness of the surface to ensure resistance to bending.

Hydra consists of two body layers, the outer ectoderm and inner endoderm. Super-cellular/Supra-cellular actin cables are present in both layers of the animal and are perpendicular to each other. That is to say that if $\hat{\underline{p}}$ describes the orientation of the cables in the ectoderm, then we can define a second vector field $\hat{\underline{q}}$, such that $\hat{\underline{p}}.\hat{\underline{q}} = 0$, which describes the orientation of the actin cables in the endoderm.

Since $\hat{\underline{p}}$ and $\hat{\underline{q}}$ are unit vectors we can write

$$\hat{\underline{p}} = \cos(\psi)\hat{\underline{x}} + \sin(\psi)\hat{\underline{y}} \quad (5)$$

$$\hat{\underline{q}} = \cos(\psi \pm \pi/2)\hat{\underline{x}} + \sin(\psi \pm \pi/2)\hat{\underline{y}}, \quad (6)$$

where ψ indicates the in-plane orientation of $\hat{\underline{p}}$ and $\hat{\underline{x}}$ and $\hat{\underline{y}}$ denote two orthogonal unit vectors in the surface's tangent plane.

When transforming between $\hat{\underline{p}}$ and $\hat{\underline{q}}$ the position and charge of topological defects is preserved, however the phase changes by $\pi/2$. For defects with charge different from 1, this indicates a rotation of the defect. For +1 topological defects a phase change of $\pi/2$ corresponds to a change between asters and vortices, and in the case of spirals to a change of the chirality.

The behaviour of the two layered system is completely captured by the introduced model if the order parameters of both layers is the same. Indeed, since the field $\hat{\underline{q}}$ is completely specified by the field $\hat{\underline{p}}$, it does not introduce any additional degrees of freedom. Explicitly, consider Eq. 1. The orientation field enters in the following term

$$\lambda(t)S(\underline{r}_i)[(\hat{\underline{p}} \cdot \hat{\underline{l}}_i)^2 - 0.5]. \quad (7)$$

Since $\hat{\underline{p}}$ and $\hat{\underline{l}}$ are unit vectors we can write this as

$$\lambda(t)S(\underline{r}_i)[\cos^2(\beta) - 0.5]. \quad (8)$$

where β is the angle between $\hat{\underline{p}}$ and $\hat{\underline{l}}$. For the second vector field $\hat{\underline{q}}$, we have analogously

$$\lambda_2(t)S(\underline{r}_i)[(\hat{\underline{q}} \cdot \hat{\underline{l}}_i)^2 - 0.5]. \quad (9)$$

Once again we can write

$$(\hat{\underline{q}} \cdot \hat{\underline{l}}_i)^2 - 0.5 = \cos^2(\beta \pm \pi/2) - 0.5 \quad (10)$$

$$= \sin^2(\beta) - 0.5 \quad (11)$$

$$= 0.5 - \cos^2(\beta) \quad (12)$$

$$= -[(\hat{\underline{p}} \cdot \hat{\underline{l}}_i)^2 - 0.5]. \quad (13)$$

Here, we have employed the angle addition formula for cos and the Pythagorean identity. Thus the sum of Eqs. 7&9 can be written as

$$[\lambda(t) - \lambda_2(t)]\, S(\underline{r}_i)[(\underline{\hat{p}} \cdot \underline{\hat{l}}_i)^2 - 0.5]. \tag{14}$$

In summary, by introducing the field $\underline{\hat{q}}$ into Eq. 1, we have merely changed the interpretation of the phenomenological parameter λ, which now is interpreted as the combination of extensions and contractions in both the endoderm and ectoderm. For this reason it is sufficient to consider in our simulations a single vector field $\underline{\hat{p}}$.

II. SIMULATION OF SPHERICAL SHELLS

All simulations performed in this paper are for thin active elastic materials, in which the thickness is much smaller than the other dimensions. We refer to these as shells. For all visualisations, the mid-plane of the thin material is approximated by coarse-graining the point cloud to a set of evenly distributed bins in the plane shell. This mid-plane of the shell is what is displayed in the images of the final configuration in all figures.

A. Preparation of the Voronoi tessellation

When simulating an initially spherical shell, the simulation is prepared as follows. First, the shell thickness is designated t in units of the spherical inner radius. Thus the volume of the simulated material is given by $V = 4\pi((1+t)^3 - 1)/3$. In this volume N points are randomly placed with uniform probability density. Finally, a single additional auxiliary point is placed at the origin, shown in red in Fig. S8a. We then generate a Delaunay triangulation from these points. After removing the auxiliary point at the origin, there are no connections across the body of the sphere.

Simulations originating from spherical shells contain 2000 points. Spherical shells are initialized with an inner radius of $R = 1$ and thickness $R/10$. We set $\lambda = 1$.

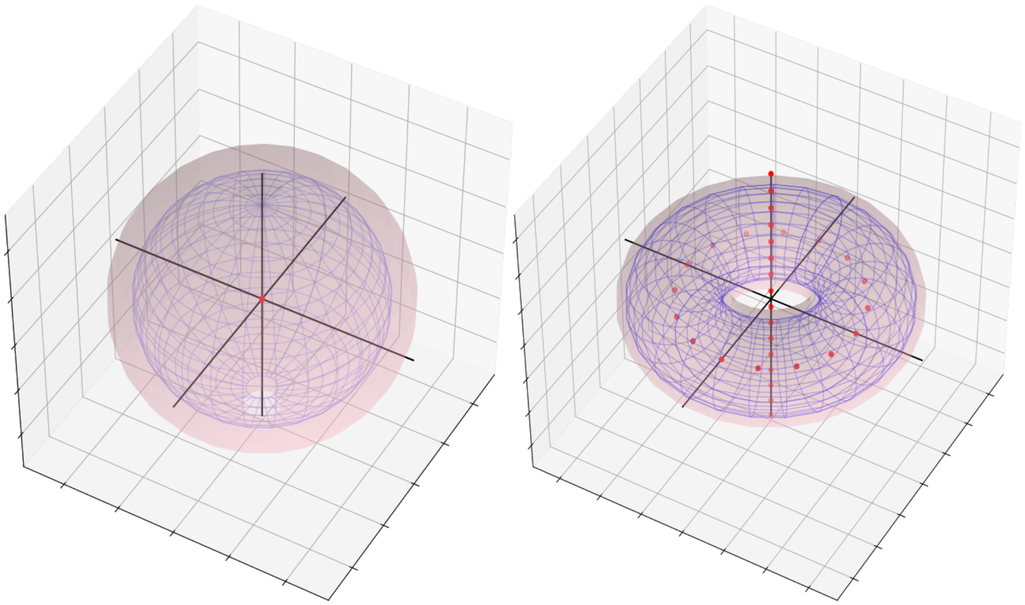

Fig. S8. Schematic of the initialisation of (a) spherical and (b) toroidal shells. The blue wireframe shows the inner surface and the pink sphere shows the outer surface. Auxiliary points are shown in red.

B. Compression of the active material

External compression of the material is captured by an additional elastic force on each node of the Delaunay triangulation that are sufficiently far from the origin of the coordinate system in the compression direction, $\underline{\hat{c}}$. The

origin of the coordinate system is centred on the centre of mass of the material. The additional restorative force experienced by particle j is given by

$$\underline{f}_j = \begin{cases} -k_c(\underline{r}_j.\hat{\underline{c}} - R_c)\hat{\underline{c}}, & \underline{r}_j.\hat{\underline{c}} > R_c \\ 0, & |\underline{r}_j.\hat{\underline{c}}| < R_c \\ k_c(\underline{r}_j.\hat{\underline{c}} - R_c)\hat{\underline{c}} & \underline{r}_j.\hat{\underline{c}}, < -R_c \end{cases}. \tag{15}$$

In our simulations, we set R_c to be $1.5R$, where R is the initial inner radius of the spherical shell, and $k_c = 1$. Compression only has a significant effect on the outcome of the simulation in the case of a non-regenerating Hydra on which it causes the +1 topological defects to invert. Compression is not included in the toroidal simulations since they correspond to Hydra post release.

C. Designation of the director field

We assume that the director field is constant across the thickness of the surface. This is described at the start of a simulation as a function of the angular position on the surface of the sphere given by the polar and azimuthal angles Θ and Φ, respectively. The director field at each point on the sphere can then be expressed by the angle it makes with the meridians of the sphere, ψ. This gives

$$\hat{\underline{p}} = \cos(\psi)\hat{\underline{g}}_\theta + \sin(\psi)\hat{\underline{g}}_\phi, \tag{16}$$

where $\hat{\underline{g}}$ are the orthonormal basis vectors on the surface of the sphere.

We calculate the orientation of the nematic field at each point using a stereographic projection to place these defects onto the complex plane $z(\Theta, \Phi) = R\cot(\Theta/2)e^{i\Phi}$, where R is the radius of the sphere. We complete the nematic field by minimising the Frank free energy.

The in-plane nematic director that minimises the Frank free energy around a set of j defects with positions z_j and charges m_j is given by $\hat{\underline{n}} = (\cos(\alpha), \sin(\alpha))$ with

$$\alpha = \alpha_0 + \sum_j \mathrm{Im}(\ln(z - z_j)^{m_j}). \tag{17}$$

Here we have introduced a global phase ψ_0. The director field is then projected back onto the sphere using $\psi(\Theta, \Phi) = \Phi - \alpha(\Theta, \Phi)$. This provides a nematic field that minimizes the elastic energy around the defects specified in the one constant approximation. Care must be taken to ensure $\sum_j m_j = 2$ consistent with the Poincaré Hopf Theorem (*32*).

The order parameter associated with the director field is given by

$$S = 1 - \sum_j \exp(-\Delta_j/\epsilon) \tag{18}$$

where Δ_j is the geodesic distance from defect j and we have introduced the defect core radius ϵ.

To predict the relative change in length of a particular line on the surface, we must perform a line integral of Eq. (1). For a path parameterized by coordinate s with tangent $\hat{\underline{t}}(s)$ and an original length L', the new path length L is given by

$$\frac{L}{L'} = \int \sqrt{1 + \zeta S(s) + \lambda S(s)[(\hat{\underline{p}}.\hat{\underline{t}})^2 - 0.5]}ds. \tag{19}$$

This shows that the extremal relative change in the length of a line segment is $\frac{L}{L'} \in [\sqrt{1 + \zeta - \lambda/2}, \sqrt{1 + \zeta + \lambda/2}]$. For the simulation values of $\zeta = 0$ and $\lambda = 1$ this gives $\frac{L}{L'} \in [\sqrt{0.5}, \sqrt{1.5}]$.

For lines of constant longitude, the tangent is given by $\hat{\underline{t}} = \hat{\underline{g}}_\theta$ and the coordinate is simply θ. Furthermore we can use the fact that

$$(\hat{\underline{p}}.\hat{\underline{g}}_\theta)^2 - 0.5 = \cos^2(\psi) - 0.5 = \cos(2\psi)/2. \tag{20}$$

This allows us to estimate the relative growth of lines of longitude as

$$\frac{L}{\pi R} = \int \sqrt{1 + \zeta S(\Theta) + 0.5\lambda S(\Theta)\cos(2\psi)}ds \tag{21}$$

which is shown in the supplementary simulation results on the positions of negative defects.

A summary of the topological defects in each simulation is included in tables. S1-S5.

TABLE S1. **Sphere with two +1 defects, compression perpendicular to the axis passing through the poles: Fig. 2C (Main text)**

Defect charge	Feature	Θ	Φ
+1	Mouth	0	0
+1	Foot	π	0
-	Compression direction	$\pi/2$	$\pi/2$

TABLE S2. **Sphere with three +1 defects, compression perpendicular to the plane containing the +1 defects: Fig. 2C (Main text)**

Defect charge	Feature	Θ	Φ
+1	Mouth 1	$\pi/3$	0
+1	Mouth 2	$\pi/3$	π
+1	Foot	π	0
-1/2	Additional defect 1	$\pi/6$	$3\pi/2$
-1/2	Additional defect 2	$\pi/6$	$\pi/2$
-	Compression direction	$\pi/2$	$\pi/2$

TABLE S3. **Sphere with two +1 defects, compression along the axis passing through the poles: Fig. 4a (Main text)**

Defect charge	Feature	Θ	Φ
+1	Mouth 1	0	0
+1	Foot	π	0
-	Compression direction	0	0

TABLE S4. **Sphere with four +1 defects, compression perpendicular to the plane containing the +1 defects: SI Fig.S9**

Defect charge	Feature	Θ	Φ
+1	Mouth	0	0
+1	Foot	π	0
+1	Tentacle 1 tip	$2\pi/8$	0
-1/2	Tentacle 1 base	$3\pi/8$	$\pi/3$
-1/2	Tentacle 1 base	$3\pi/8$	$5\pi/3$
+1	Tentacle 2 tip	$2\pi/8$	π
-1/2	Tentacle 2 base	$3\pi/8$	$2\pi/3$
-1/2	Tentacle 2 base	$3\pi/8$	$4\pi/3$
-	Compression direction	$\pi/2$	$\pi/2$

For completeness, we also include a spherical surface with topological defects associated with the tentacles on the Hydra. In keeping with the topological constraints, each tentacle has a +1 defect at its tip and a pair of $-1/2$ defects at its base. This makes them each topologically neutral and thus the total number of tentacles is not topologically constrained.

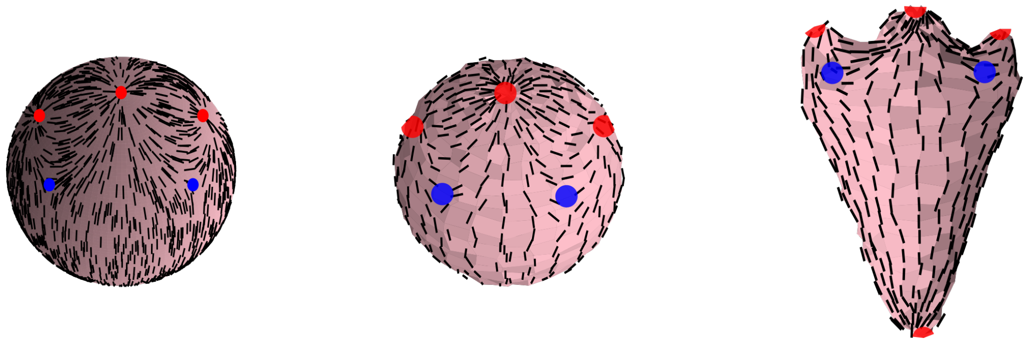

Fig. S9. Surface evolving with the topological defects associated with a Hydra featuring two tentacles. Left) Nematic texture around the specified defects. Middle) Starting point of the simulation. Right) End point of the simulation.

III. SIMULATION OF TOROIDAL SHELLS

A. Preparation of the Voronoi tessellation

When simulating an active elastic material with a toroidal topology, the simulation is prepared as follows. First, the shell thickness is designated t in units of the inner, minor radius of the torus. Thus the volume of the simulated material is given by $V = 2\pi^2((1+t)^2 - 1)$. In this volume N points are randomly placed with uniform probability density.

Due to the more complex topology of the torus, a set of additional auxiliary points are placed in and around the torus. These are located on a ring inside the torus, and on a line that threads the centre of the torus, Fig. S8b. We then generate a Delaunay triangulation including these points. The auxiliary points in and around the torus are removed before the simulation begins and thus ensure that the hollow regions of the simulation are not connected. Toroidal simulations are initialised with 10000 points, an inner minor radius of $R = 1$, and a thickness of $R/10$. We set $\lambda = 1.5$.

B. Designation of the director field on the torus

We assume that the director field is constant across the thickness of the material, such that it can be described as a function of the position on the surface of the torus given by the curvilinear coordinates Θ and Φ, which designate the

TABLE S5. **Torus with two +1 defects: Fig.4c (Main text)**

Defect charge	Feature	Θ	Φ
+1	Mouth	0	0
+1	Foot	0	π
-1/2	Additional defect 1	0	$3\pi/2$
-1/2	Additional defect 2	0	$\pi/2$
-1/2	Additional defect 3	π	0
-1/2	Additional defect 4	π	π

poloidal and toroidal directions, respectively. Without loss of generality we assign $\Theta = 0$ to denote the outer equator of the torus.

The director field at each point on the sphere can then be given by the angle it makes with the meridians of the torus, ψ. This gives

$$\underline{\hat{p}} = \cos(\psi)\underline{\hat{g}}_\theta + \sin(\psi)\underline{\hat{g}}_\phi \tag{22}$$

Where $\underline{\hat{g}}$ are the orthonormal basis vectors on the surface of the torus.

For simulations corresponding to non-regenerating toroidal Hydra, we set $\psi = 0$ everywhere as is observed in experiments, such that it features no defects.

For simulations corresponding to toroidal Hydra with delayed regeneration, we use a director field with defects. To generate the orientation field around a set of defects, first we generate the field on flat surface with periodic boundaries. The director field is given by

$$\psi = \sum_j k_j \phi_j, \tag{23}$$

where k_j is the charge and ϕ_j is the polar angle relative to defect j, respectively. A smoothly varying phase may be added to this field to ensure the desired phase of the defect cores.

The order parameter associated with the director field is calculated as

$$S = 1 - \sum_j \exp(-\Delta_j/\epsilon), \tag{24}$$

where Δ_j is the Euclidian distance from defect j and we have introduced the defect core radius ϵ.

Finally, the Landau-de Gennes free energy of the field is minimised iteratively in regions for which $S > S_c$. This cut-off for S is to fix the position and orientation of the defects, otherwise they would migrate during energy minimisation.

The defect positions for recovering toroidal hydra are given in Table. S5. The non-recovering toroidal hydra features no topological defects.
